# Supplementary material for: EGOC inhibits TOROID polymerization by structurally activating TORC1
Source: Nat Struct Mol Biol. 2023 Jan 26;30(3):273–85. doi: 10.1038/s41594-022-00912-6 (PMC10023571; doi:10.1038/s41594-022-00912-6)
Supplement: Supplementary file 1 — List of strains, plasmids and oligonucleotides. [file 41594_2022_912_MOESM1_ESM.pdf]

nature structural & molecular biology

Article https://doi.org/10.1038/s41594-022-00912-6

EGOC inhibits TOROID polymerization by structurally activating TORC1

In the format provided by the authors and unedited

Supplementary Table 1: List of strains used in this study

S. ceRisiae: TB50: JK9-3da MATa leu2-3,112 ura3-52 trp1 his3 rme1 HMLa	[1]
S. ceRisiae: BY4741:MATa *his3Δ1 leu2Δ0 met15Δ0 ura3Δ0*	[2]
RL3823: MATa; TB50, *gtr1Δ*::TRP1, *gtr2Δ*::HPH, *VPH1::mCherry-KanMX*4	This study
RL3013: MATα; TB50, LEU2::*GFP-TOR1*	[3]
RL3788: TB50, LEU2::*GFP-TOR1, lst4::*KanMX6	This study
RL3832: TB50, LEU2::*GFP-TOR1, gtr1Δ::TRP1, gtr2Δ::HPH lst4::*KanMX6	This study
RL3789: TB50, LEU2::*GFP-TOR1, lst7::*NatMX4	This study
RL3826: TB50, LEU2::*GFP-TOR1,* sea1*::*KanMX6	This study
TB50, LEU2*::GFP-TOR1* $LST8^{Q29A}$	This study
TB50, LEU2::*GFP-TOR1* $LST8^{H292A}$	This study
TB50, LEU2::*GFP-TOR1* $KOG1^{\Delta\alpha21,\ 725\text{-}736(AG)n}$	This study
TB50, LEU2::*GFP-TOR1* $KOG1^{HC,\ (KOG1(802\text{-}905)::Raptor(624\text{-}653))}$	This study
TB50, LEU2::*GFP-TOR1* $KOG1^{\Delta twix\ (807\text{-}881)}$	This study
TB50, LEU2::*GFP-TOR1* $KOG1^{Screen\ (717\text{-}895)}$	This study
TB50, LEU2::*GFP-TOR1* $KOG1^{\Delta tack(1068\text{-}1086)}$	This study
TB50, LEU2::*GFP-TOR1* $KOG1^{\Delta 1004\text{-}22}$	This study
TB50, LEU2::*GFP-TOR1* $KOG1^{\Delta claw\ (1121\text{-}1132)}$	This study
TB50, LEU2::*GFP-TOR1* $KOG1^{E784A}$	This study
TB50, LEU2::*GFP-TOR1* $KOG1^{R895\text{-}896A}$	This study
TB50, LEU2::GFP-*TOR1* $KOG1^{R1383A}$	This study
TB50, LEU2::GFP-*TOR1* $KOG1^{\Delta 1544\text{-}end}$	This study
TB50, LEU2::GFP-$TOR1^{326\text{-}334(AG)n}$	This study
TB50, LEU2::GFP-$TOR1^{368\text{-}370(AG)n}$	This study
TB50, LEU2::*GFP*-$TOR1^{W1279A}$	This study
TB50, LEU2::*GFP*-$TOR1^{1449/54/56A}$	This study
TB50, LEU2::*GFP*-$TOR1^{W2203R}$	This study
TB50, LEU2::*GFP-TOR1* $TOR2^{337\text{-}345(AG)n}$	This study
TB50, LEU2::*GFP-TOR1,*$TOR2^{379\text{-}381(AG)n}$	This study
TB50, LEU2*::GFP-TOR1,* $TOR2^{K2053/53F}$	This study
TB50, LEU2::*GFP-TOR1,* $TOR2^{W2207R}$	This study
TB50, LEU2::*GFP*-$TOR1^{W2203R}$, $TOR2^{W2207R}$	This study
TB50, LEU2::*GFP*-$TOR1^{W2203R}$, $TOR2^{W2207R}$, $KOG1^{HC}$	This study
RL3821: TB50, *LEU2::GFP-TOR1, ego1Δ*::HIS3	This study
RL3015: MATa; TB50, LEU2::*GFP-TOR1, gtr1Δ::*TRP1*, gtr2Δ::*HPH	[3]
TB50, $EGO1^{TEV}$ *, LEU2::GFP-TOR1*, pRS416	This study
TB50, $EGO1^{TEV}$ *, LEU2::GFP-TOR1*, pRS416: P_{CTH2}-6xHIS-TEV-T_{CTH2}	This study
SKY596: MATa, BY4741, LEU2::*GFP-TOR1*, *EGO3::3xmCherry-NatMX4*	[4]
RL2500: MATα; TB50, *GFP-KOG1*	[3]
RL3867: TB50, GFP-KOG1, *gtr1Δ*::TRP1, *gtr2Δ*::HPH	[3]
RL3843: TB50, *GFP-KOG1 EGO3::3xmCherry-NatMX4*	This study

RL3872: TB50, *GFP-KOG1 EGO3::3xmCherry-NatMX4* , *gtr1Δ*::TRP1, *gtr2Δ*::HPH	This study
RL3642: MATα; TB50, *EGO3::GFP-HPH*	This study
RL3786: TB50, EGO3::GFP-HPH, *lst4::*KanMX6	This study
RL3833: TB50, EGO3::GFP-HPH, *gtr1Δ::TRP1, gtr2Δ::HPH lst4::*KanMX6	This study
RL3787: TB50, EGO3::GFP-HPH, *lst7::*NatMX4	This study
RL3827: TB50, EGO3::GFP-HPH, sea1*::*KanMX6	This study
RL3816: TB50, $EGO1^{TEV}$ *EGO3::GFP-HPH*	This study
RL3824: TB50, *EGO3::GFP-HPH, ego1Δ*::HIS3	This study
RL3734: TB50, *EGO3::GFP-HPH*, *gtr1Δ*::TRP1, gtr2Δ::HPH	This study
RL171-2d: MATa; TB50,*KOG1::TAP-HIS3*	[3]
RL174-5b: MATa; TB50, *KOG1::TAP-HIS3 tor1Δ::*KanMX6	This study
RL3692: TB50, $KOG1^{HC}$*::TAP-KanMX6*	This study

Supplementary Table 2: List of plasmids used in this study

pRS415: *CEN/ARS, LEU2*	[5]
pRS416: *CEN/ARS, URA3*	[5]
pRS416: P_{CTH2}-*3xHA-TEV*-T_{CTH2}	This study
pRS416: P_{CTH2}-*6xHIS-TEV*-T_{CTH2}	This study
pRS415: *GTR1*	[6]
pRS415: $GTR1^{Q65L}$ (expressing $Gtr1^{GTP}$ locked)	[6]
pRS415: $GTR1^{S20L}$ (expressing $Gtr1^{GDP}$ locked)	[6]
pRS416: *GTR2*	[6]
pRS416: $GTR2^{Q66L}$ (expressing $Gtr2^{GTP}$ locked)	[6]
pRS416: $GTR2^{S23L}$ (expressing $Gtr2^{GDP}$ locked)	[6]
pACYC-T7: $EGO2^{\Delta1\text{-}21}$-*EGO3-6xHIS* -$EGO1^{\Delta1\text{-}111}$(codon optimized)	This study
pET-42 w/out tag: *GTR1-GTR2*	This study
pET-42 w/out tag: $GTR1^{Q65L}$-$GTR2^{S23L}$	This study
pET-42 w/out tag: $GTR1^{S20L}$-$GTR2^{Q66L}$	This study

Supplementary Table 3: List of oligonucleotides used for plasmid design and CrispR/Cas9 mutageneses.

Target		Oligonucleotide sequence
pML104	F	GATCATTTATCTTTCACTGCGGAGAAGT
	R	GTTTTAGAGCTAGAAATAGCAAGTTAAAATAAGGCTAGTCCGT
$LST8^{65}$	F	gcagtgaaagataaatgatcATTTTGGGAGGCTCTTACTGgttttagagctagaaatagc
	R	gctatttctagctctaaaacCAGTAAGAGCCTCCCAAAATgatcatttatctttcactgc
$LST8^{878}$	F	gcagtgaaagataaatgatcACAGTACGGTGGACACCACAgttttagagctagaaatagc
	R	gctatttctagctctaaaacTGTGGTGTCCACCGTACTGTgatcatttatctttcactgc
$KOG1^{2186}$	F	gcagtgaaagataaatgatcATTCGTTCGAAACTTTCCTCgttttagagctagaaatagc
	R	gctatttctagctctaaaacGAGGAAAGTTTCGAACGAATgatcatttatctttcactgc
	F	gcagtgaaagataaatgatcGTTGTAGATGGGATTGATTTgttttagagctagaaatagc

KOG1 2293	R	gctatttctagctctaaaacAAATCAATCCCATCTACAACgatcatttatctttcactgc
KOG1 2340	F	gcagtgaaagataaatgatcAGGTTTGTATGTATGAATACgttttagagctagaaatagc
	R	gctatttctagctctaaaacGTATTCATACATACAAACCTgatcatttatctttcactgc
KOG1 2666	F	gcagtgaaagataaatgatcTTTGAGGAAGTTAAAAAGACgttttagagctagaaatagc
	R	gctatttctagctctaaaacGTCTTTTTAACTTCCTCAAAgatcatttatctttcactgc
KOG1 4147	F	gcagtgaaagataaatgatcTGGAATCTCTTGGATCGAGAgttttagagctagaaatagc
	R	gctatttctagctctaaaacTCTCGATCCAAGAGATTCCAgatcatttatctttcactgc
TOR1 991	F	gcagtgaaagataaatgatcGTCGAACACTTGATTCAAAAgttttagagctagaaatagc
	R	gctatttctagctctaaaacTTTTGAATCAAGTGTTCGACgatcatttatctttcactgc
TOR1 1112	F	gcagtgaaagataaatgatcTATTTGCCAGCAAATAATTGgttttagagctagaaatagc
	R	gctatttctagctctaaaacCAATTATTTGCTGGCAAATAgatcatttatctttcactgc
TOR1 3835	F	gcagtgaaagataaatgatcATACCGCATTCGCATGTGTTgttttagagctagaaatagc
	R	gctatttctagctctaaaacAACACATGCGAATGCGGTATgatcatttatctttcactgc
TOR1 4345	F	gcagtgaaagataaatgatcCGCAATTGGCAGCTAGAAAGgttttagagctagaaatagc
	R	gctatttctagctctaaaacCTTTCTAGCTGCCAATTGCGgatcatttatctttcactgc
TOR1 6623	F	gcagtgaaagataaatgatcACATTGGGTTATGTTACAAAgttttagagctagaaatagc
	R	gctatttctagctctaaaacTTTGTAACATAACCCAATGTgatcatttatctttcactgc
TOR2 1025	F	gcagtgaaagataaatgatcTCATATTTATCCCTGAGATAgttttagagctagaaatagc
	R	gctatttctagctctaaaacTATCTCAGGGATAAATATGAgatcatttatctttcactgc
TOR2 1144	F	gcagtgaaagataaatgatcATTTCTTTGTGAAAATGGCAgttttagagctagaaatagc
	R	gctatttctagctctaaaacTGCCATTTTCACAAAGAAATgatcatttatctttcactgc
TOR2 6153	F	gcagtgaaagataaatgatcTATAATGTTTTCAGGAAAATgttttagagctagaaatagc
	R	gctatttctagctctaaaacATTTTCCTGAAAACATTATAgatcatttatctttcactgc
TOR2 6619	F	gcagtgaaagataaatgatcTTCCTTTAAACATTGAGCATgttttagagctagaaatagc
	R	gctatttctagctctaaaacATGCTCAATGTTTAAAGGAAgatcatttatctttcactgc
Lst8^{Q} 29A	F	TCTGCTGGCTATGACCACACAATAAGATTTTGGGAGGCTGCAGGAGGTGTGTGCTCGAGGACGATCGCA
	R	GTTGCCAACAACTTTTTATCGTTGGTGATTTCTAAGCGATTAACCTGTGAGCCGGCTCCTGCGATCGTCCTCGAGCAC
Lst8^{H} 292A	F	TGAGATTATGGGATTTATCAACTAGAGAAATTGTCAGACAGTACGGTGGAGCGGGAGCCGGA
	R	AGTTGATAAATTGTAAACTATACATCGTTTAATGCGACACATACAGCTCCGGCTCCCGCTCC
Kog1 Δα21	F	TAATGATACCACCGATGAACAAAAAGCTATGGCTGTCTTTGTTTTGGCATCATTCGTTCGAGGAGCAGGTGCTGGCT
	R	TCTTAGTAGGGGTATCTCAGAATTATCAATATAAAAGCACAACTTGTTGACCAGTTCCAAGCCAGCACCTGCTCCT
Kog1 HT	F	CTTTGAAGGACCCTGTACCTGAAGTTAGAACAGCTTCTATATTTGCATTGGGTACTTTTGTAGGTAATAGTGCAGAACGTACTGACAGCACTACAATTGATCACAACG
	R	ATATGCGAAAAATATACGACTAGTTCCTTACGGACTAATGATGAACCATCTGAAACAAGTTGTGCAAGCATCATGGCTACGTTGTGATCAATTGTAGTGCTGTCAG
Kog1 Δtwix	F	ACCTGAAGTTAGAACAGCTTCTATATTTGCATTGAAGCATTTCATATCTGGCGCAGGAGCTGGTGCAGG
	R	AAGATTGAGATCAAGTTGCCAATTTCCTGTCTTTTTAACTTCCTCAAATCTGCACCTGCACCAGCTCCTGC

Kog1^{Screen}	F	AATAATCTTCCATTTTATCATTCTAATGATACCACCGATGAACAAAAAGCTATGGCTGTCTTTGTTCTGGCGAGCTTCGT
	R	ACGACTCACAATATGCGAAAAATATACGACTAGTTCCTTACGGACTAATGATGAACCATCGTTGATCAGCGGCAGAATGCTG
Kog1 Δtack	F	AGATGCAGAGGTAATTTTGAGATTGCAACAAGAATTTGAAGAACAGTATCAGCAATTACATAGCCAGCTGCAACACCTG
	R	CTTTTTTGAGGTATGCGATTTCATCGAAGTATTTGATGATTGAGTATCAGAGTCACTATTTCCTGCCCCCGCGCCGGCACCAGCTCCTGCCCCAGCGCCTGCCCCGGCACCTGCGTGGCTAATTTTCGGATCGTGTTG
Kog1 Δ1004-22	F	AGATGCAGAGGTAATTTTGAGATTGCAACAAGAATTTGAAGAACAGTATCAGCAATTACATAGCCAGCTGCAACACCTG
	R	TCGTAATGAACTTTTTACAAATTGAACCTGCGAAGAATTAAATCCAAATTTACCAGTTTGTCCTGCCCCCGCGCCGGCACCAGCTCCTGCCCCAGCGCCTGCCCCGGCACCTGCACCCAGTTCCTTGTGCGC
Kog1 Δclaw1	F	GCTTATATTTATTGAATGGCAATAATAATATTTATCCTACAGCTGAGACTGGCGCAGGAGCTGGTGC
	R	GTGAATAGTCTAAAAAGGAACTATTTAATGGCAATTGTAAGGGTTCTGTTGCACCAGCTCCTGCGC
Kog1 Δclaw2	F	AGATGCAGAGGTAATTTTGAGATTGCAACAAGAATTTGAAGAACAGTATCAGCAATTACATAGCCAGCTGCAACACCTG
	R	AAAATATTCACGTGAATAGTCTAAAAAGGAACTATTTAATGGCAATTGTAAGGGTTCTGTAGCGCCTGCACCCGCTCCAGCGCCGGCACCGGCATAGATGTTGTTGTTGCCGTTCAGC
Kog1^{E784A}	F	AGTGGTGTGTTATTTTATTAGGTTTATTGTTTGCTGATAACCCCTTAAACCGTTTCGTCTGCATGAACACTGGCGCGGTGGC
	R	ATGCTTCAATGCAAATATAGAAGCTGTTCTAACTTCAGGTACAGGGTCCTTCAAAGATTTTAGTAATATTGCCACCGCGCCAGT
Kog1 R895-896A	F	AGTTATGCAAAATCAACTAGAAGTTATTGATTTGAGGAAGTTAAAAAGACAAGAGATCGGTAATCTGGCCGC
	R	ACTAGTTCCTTACGGACTAATGATGAACCATCATTGATTAAAGGTAAGATTGCGGCCAGATTACCGATCTCT
Kog1 R1383A	F	AATATCTTCGTAGCAGGGTTTGCAGATGGCTCCCTGAGAGTATATGATCGCAGATTGGACCCTGCTGCA
	R	ATCCAAACTCCCTGCTTATCATTTCCAGCTCTCCAGCGACGTATCATGGATGCAGCAGGGTCCAATCTG
Tor1$^{326-334(AG)}$	F	AACACATTAGAATGCATCCATGCAAGTTTGTTGGTTTATAAGGAAATCTTGGGGCTGGAGCAGGCGCTGGTGCAGGA
	R	CTTTATGATTTTCATAAGCTATGCAATTTAGACACATTTGGTCGAACACTTCCTGCACCAGCGCCT
Tor1$^{368-370(AG)}$	F	AAGCGAAAATGATTAGAGAAAAGATTTACCAGATTGTTCCCCTATTAGCAGCGGGCACTCCTCAGTTGT
	R	TTGGTTAAAATCTCTAAATAGTTGTCCATAATTTGGTGCAAATATTTGCCCGCGAACAACTGAGGAGTGCCCGCT
Tor1 W1279A	F	CAAATCTTGCAAGCATGTATTATCCACTAGCCAAAGAACTTTTTAATACCGCCTTTGCCTGCGTCGCGAC
	R	CTATACATAATGACCCAATTAAATCTTCTTGATATTGGCTATAAAGTTCGGTCGCGACGCAGGCAAAGGC
Tor1$^{1449/54/56A}$	F	TGAGATCCCTTCATGCCCTTGGCGAATGGGAACAGTTGTCGCAATTGGCGGCGAGGAAAGCCAAAGTTTCGA
	R	CCACGCAGCACCAGCTGCCAAGGGAGCTATTAGCTTCTTAGCTTGTGCCTTCGAAACTTTGGCTTTCCTCGC
Tor1 W2203R	F	GGGTACCAAATAGTGACACATTCCACGTTTTGATCAGAGAACACCGTGATGCCAAAAAAATCCCGCTGAACATCGAGCAC
	R	TACTTCAATTTTTTGTAAAAGAGTCAAATTCTCATAATCGGGGGCCATTTGTAACATAACGCGGTGCTCGATGTTCAGCGGGA

Tor2$^{337-345(AG)}$	F	TCAATACGAATGATTCAGTGCATGCTACTCTGTTGGTATTTCGAGAATTAGCTGCAGGCGCAGCTGGTGCA
	R	TTATATTCCTTGTACTTCATAGTAGATTTGTAAATATCATCATATTTATCTCCAGCTGCACCAGCTGCGCC
Tor2$^{379-381(AG)}$	F	TTGATGTTATAAGGAGAGAAGTTTATGCTATTTTACCTCTTTTAGCTGCTGCTGCTCCTGCCATTTTCAC
	R	AAATAATGAACCATTATCCTATCGAGATATTTCTTTGTGAAAATGGCAGGAGCAGC
Tor2$^{K2053/53F}$	F	GCTTACGAATGGCTGATGAATTACAAAAAATCTAAAGATGTTAGTAATTTAAACCAAGCGTGAGGTTTAT
	R	AGTTTTGGCGACACATGTTGTAGTTCAAGAGTTTGTAATTGTGGCAACTGAAAACCAATAAACCTCACGC
Tor2 W2207R	F	GGGTACCGAATAGTGACACGTTCCATGTATTAATTAGGGAGCATAGAGAAGCCAAAAAAATCCCCTTGAACATCGAGCAC
	R	GACTTCTACTTTCTGCAACAACGTTAAATTGTCATAATCAGGTGCCATTTGTAACATGACGCGGTGCTCGATGTTCAAGGGGA

1. Heitman, J., Movva, N.R. & Hall, M.N. Targets for cell cycle arrest by the immunosuppressant rapamycin in yeast. *Science* **253**, 905-9 (1991).
2. Brachmann, C.B. et al. Designer deletion strains derived from Saccharomyces ceRisiae S288C: a useful set of strains and plasmids for PCR-mediated gene disruption and other applications. *Yeast* **14**, 115-32 (1998).
3. Prouteau, M. et al. TORC1 organized in inhibited domains (TOROIDs) regulate TORC1 activity. *Nature* **550**, 265-269 (2017).
4. Ukai, H. et al. Gtr/Ego-independent TORC1 activation is achieved through a glutamine-sensitive interaction with Pib2 on the vacuolar membrane. *PLoS Genet* **14**, e1007334 (2018).
5. Sikorski, R.S. & Hieter, P. A system of shuttle vectors and yeast host strains designed for efficient manipulation of DNA in Saccharomyces ceRisiae. *Genetics* **122**, 19-27 (1989).
6. Binda, M. et al. The Vam6 GEF controls TORC1 by activating the EGO complex. *Mol Cell* **35**, 563-73 (2009).
