# Supplementary material for: EGOC inhibits TOROID polymerization by structurally activating TORC1
Source: Nat Struct Mol Biol. 2023 Jan 26;30(3):273–85. doi: 10.1038/s41594-022-00912-6 (PMC10023571; doi:10.1038/s41594-022-00912-6)
Supplement: Supplementary file 2 — Reporting Summary [file 41594_2022_912_MOESM2_ESM.pdf]

nature portfolio

Corresponding author(s): LOEWITH R., PROUTEAU M., FELIX J.

Last updated by author(s): Oct 24, 2022

Reporting Summary

Nature Portfolio wishes to improve the reproducibility of the work that we publish. This form provides structure for consistency and transparency in reporting. For further information on Nature Portfolio policies, see our Editorial Policies and the Editorial Policy Checklist.

Statistics

For all statistical analyses, confirm that the following items are present in the figure legend, table legend, main text, or Methods section.

n/a	Confirmed	
☐	☒	The exact sample size (n) for each experimental group/condition, given as a discrete number and unit of measurement
☐	☒	A statement on whether measurements were taken from distinct samples or whether the same sample was measured repeatedly
☐	☒	The statistical test(s) used AND whether they are one- or two-sided<br>*Only common tests should be described solely by name; describe more complex techniques in the Methods section.*
☐	☒	A description of all covariates tested
☒	☐	A description of any assumptions or corrections, such as tests of normality and adjustment for multiple comparisons
☐	☒	A full description of the statistical parameters including central tendency (e.g. means) or other basic estimates (e.g. regression coefficient) AND variation (e.g. standard deviation) or associated estimates of uncertainty (e.g. confidence intervals)
☐	☒	For null hypothesis testing, the test statistic (e.g. F, t, r) with confidence intervals, effect sizes, degrees of freedom and P value noted<br>*Give P values as exact values whenever suitable.*
☒	☐	For Bayesian analysis, information on the choice of priors and Markov chain Monte Carlo settings
☒	☐	For hierarchical and complex designs, identification of the appropriate level for tests and full reporting of outcomes
☐	☒	Estimates of effect sizes (e.g. Cohen's d, Pearson's r), indicating how they were calculated

Our web collection on statistics for biologists contains articles on many of the points above.

Software and code

Policy information about availability of computer code

Data collection	Live-cell microscopy images were acquired either with LSM800 confocal microscope (Plan-Apochromat 63x/1.4 Oil Objective, Zeiss) or a TCS SP5 confocal microscope (Plan-Apochromat 63x/1.4 Oil Objective, Leica). Western blots were acquired with an Odyssey imager from a Li-Cor Odyssey 9120 imager. Cryo-EM images were collected on a Titan Krios microscope (Thermo Scientific), operated at 300 kV and equipped with a K2 summit direct electron detector (Gatan) camera operated in counting mode using SerialEM.
Data analysis	Western blot analysis: Software Image Studio version 5.2.5 from LI-COR; Light microscopy images processing: FIJI (ImageJ), Imaris version 9.8; Curve fit analysis: Prism 9.4.1 ; Cryo-EM: RELION 2.0, CTFFIND4, MotionCor2, EMAN2, Gctf, cryoSPARC 3.01; Structure analysis and vizualisation: USCF Chimera, USCF ChimeraX, PyMol 2.3; Model Building: DeepEMhancer, Alphafold.

For manuscripts utilizing custom algorithms or software that are central to the research but not yet described in published literature, software must be made available to editors and reviewers. We strongly encourage code deposition in a community repository (e.g. GitHub). See the Nature Portfolio guidelines for submitting code & software for further information.

Data

Policy information about availability of data

All manuscripts must include a data availability statement. This statement should provide the following information, where applicable:

- Accession codes, unique identifiers, or web links for publicly available datasets
- A description of any restrictions on data availability
- For clinical datasets or third party data, please ensure that the statement adheres to our policy

All light microscopy, western blots, spotassays and statistical analysis data are available at : 10.26037/yareta:kr6oskrjsneh5m2orna4zbqvo4.

Human research participants

Policy information about studies involving human research participants and Sex and Gender in Research.

Reporting on sex and gender	*Use the terms sex (biological attribute) and gender (shaped by social and cultural circumstances) carefully in order to avoid confusing both terms. Indicate if findings apply to only one sex or gender; describe whether sex and gender were considered in study design whether sex and/or gender was determined based on self-reporting or assigned and methods used. Provide in the source data disaggregated sex and gender data where this information has been collected, and consent has been obtained for sharing of individual-level data; provide overall numbers in this Reporting Summary. Please state if this information has not been collected. Report sex- and gender-based analyses where performed, justify reasons for lack of sex- and gender-based analysis.*
Population characteristics	*Describe the covariate-relevant population characteristics of the human research participants (e.g. age, genotypic information, past and current diagnosis and treatment categories). If you filled out the behavioural & social sciences study design questions and have nothing to add here, write "See above."*
Recruitment	*Describe how participants were recruited. Outline any potential self-selection bias or other biases that may be present and how these are likely to impact results.*
Ethics oversight	*Identify the organization(s) that approved the study protocol.*

Note that full information on the approval of the study protocol must also be provided in the manuscript.

Field-specific reporting

Please select the one below that is the best fit for your research. If you are not sure, read the appropriate sections before making your selection.

☒ Life sciences ☐ Behavioural & social sciences ☐ Ecological, evolutionary & environmental sciences

For a reference copy of the document with all sections, see nature.com/documents/nr-reporting-summary-flat.pdf

Life sciences study design

All studies must disclose on these points even when the disclosure is negative.

Sample size	No statistical methods were performed to predetermine the sample size, but the number of replicates for each experiment was based on our previous experience (Prouteau et al., 2017). Detailed information for the individual experiments including sample size and replicates are stated in the figure legends. For cryoEM studies, the number of particles used for each of the EM reconstructions has been stated in the methods section.
Data exclusions	For Cryo-EM, particles were sorted and selected as described in the "Methods" section.
Replication	At least triplicated experiments have been done for each data collection. all attempts at replication were successful.
Randomization	For cryoEM, extracted particles were randomly assigned to two separate groups to calculate half-maps and gold-standard FSC.
Blinding	All light microscopy experiments have been measured by blinded expimentators when it required manual counting. Automated measurements based on software were not blinded. For other experiments: no grouped samples.

Reporting for specific materials, systems and methods

We require information from authors about some types of materials, experimental systems and methods used in many studies. Here, indicate whether each material, system or method listed is relevant to your study. If you are not sure if a list item applies to your research, read the appropriate section before selecting a response.

Materials & experimental systems

n/a	Involved in the study
☐	☒ Antibodies
☒	☐ Eukaryotic cell lines
☒	☐ Palaeontology and archaeology
☒	☐ Animals and other organisms
☒	☐ Clinical data
☒	☐ Dual use research of concern

Methods

n/a	Involved in the study
☒	☐ ChIP-seq
☒	☐ Flow cytometry
☒	☐ MRI-based neuroimaging

Antibodies

Antibodies used

Mouse monoclonal anti-Sch9 p-S758 antibody, rabbit polyclonal anti-Sch9 antibody, mouse monoclonal anti-HA clone HA-7, ascites fluid (Sigma cat#H9658), mouse monoclonal anti-polyHistidine antibody, clone HIS-1 (Sigma cat#H1029-2ML), rabbit polyclonal anti-TAP Antibody (Open Biosystems cat#CAB1001).

Validation

Both Sch9 antibodies were made and already validated by our lab (Gaubitz et al., 2015; Prouteau et al., 2018). Anti-TAP and anti-HA antibodies were validated in papers from our lab (Binda et al., 2009; Bourgoint et al., 2018). Anti-polyhistidine antibody has been validated in numerous papers including Rodríguez-Escudero et al., 2018.
