# Supplementary figures and images for: EGOC inhibits TOROID polymerization by structurally activating TORC1
Source: Nat Struct Mol Biol. 2023 Jan 26;30(3):273–85. doi: 10.1038/s41594-022-00912-6 (PMC10023571; doi:10.1038/s41594-022-00912-6)

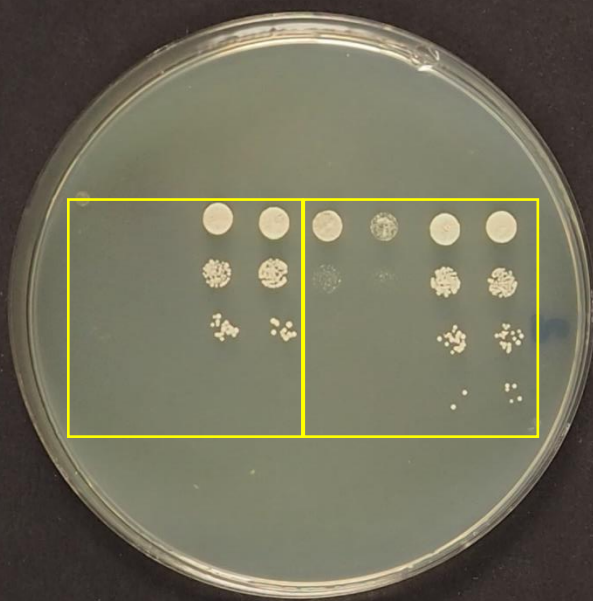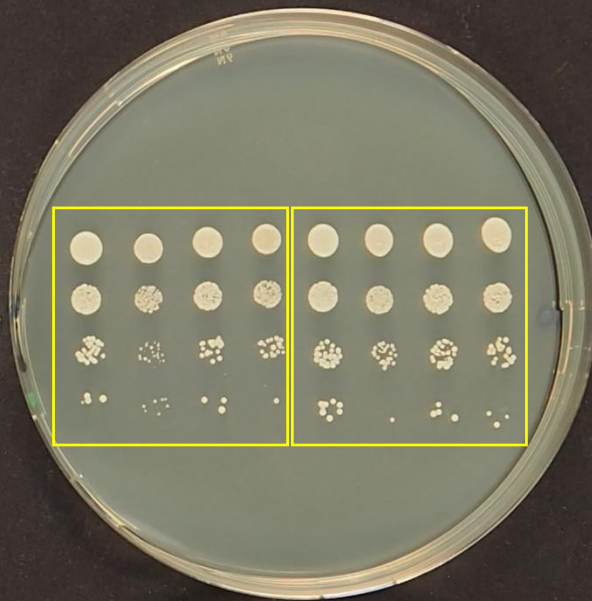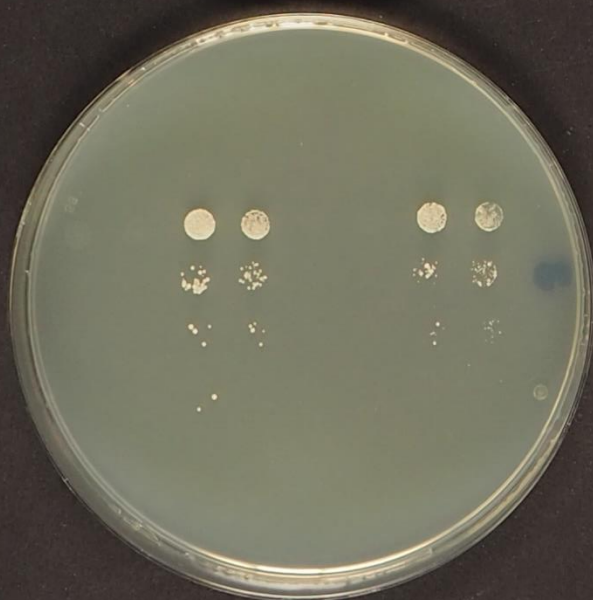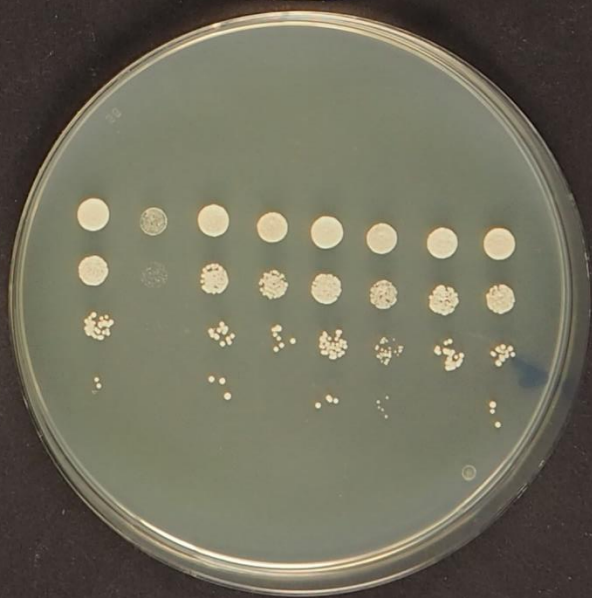

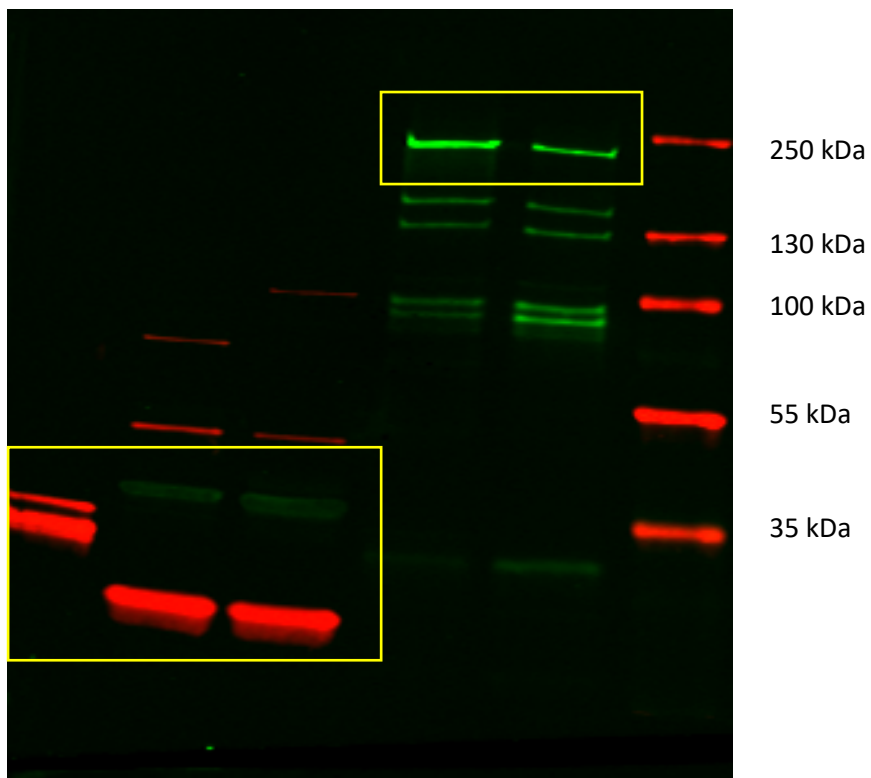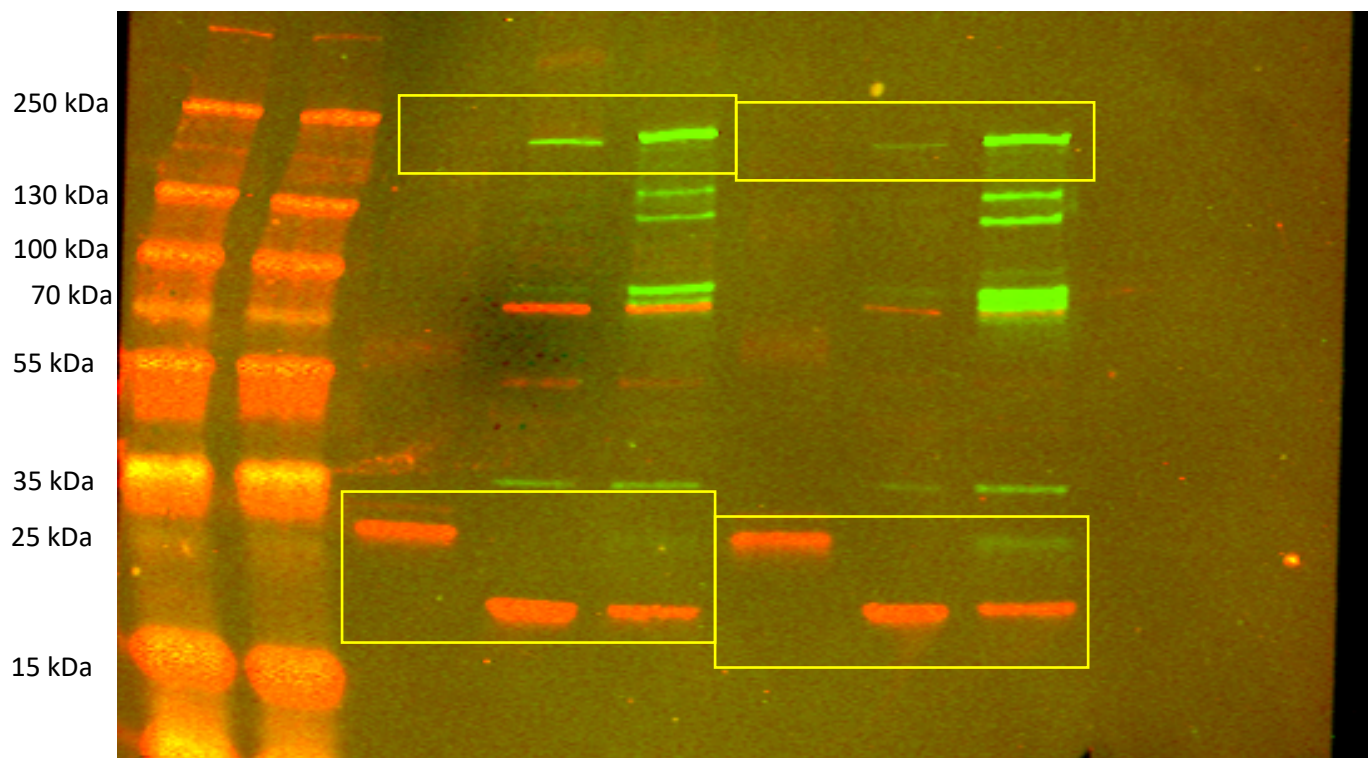

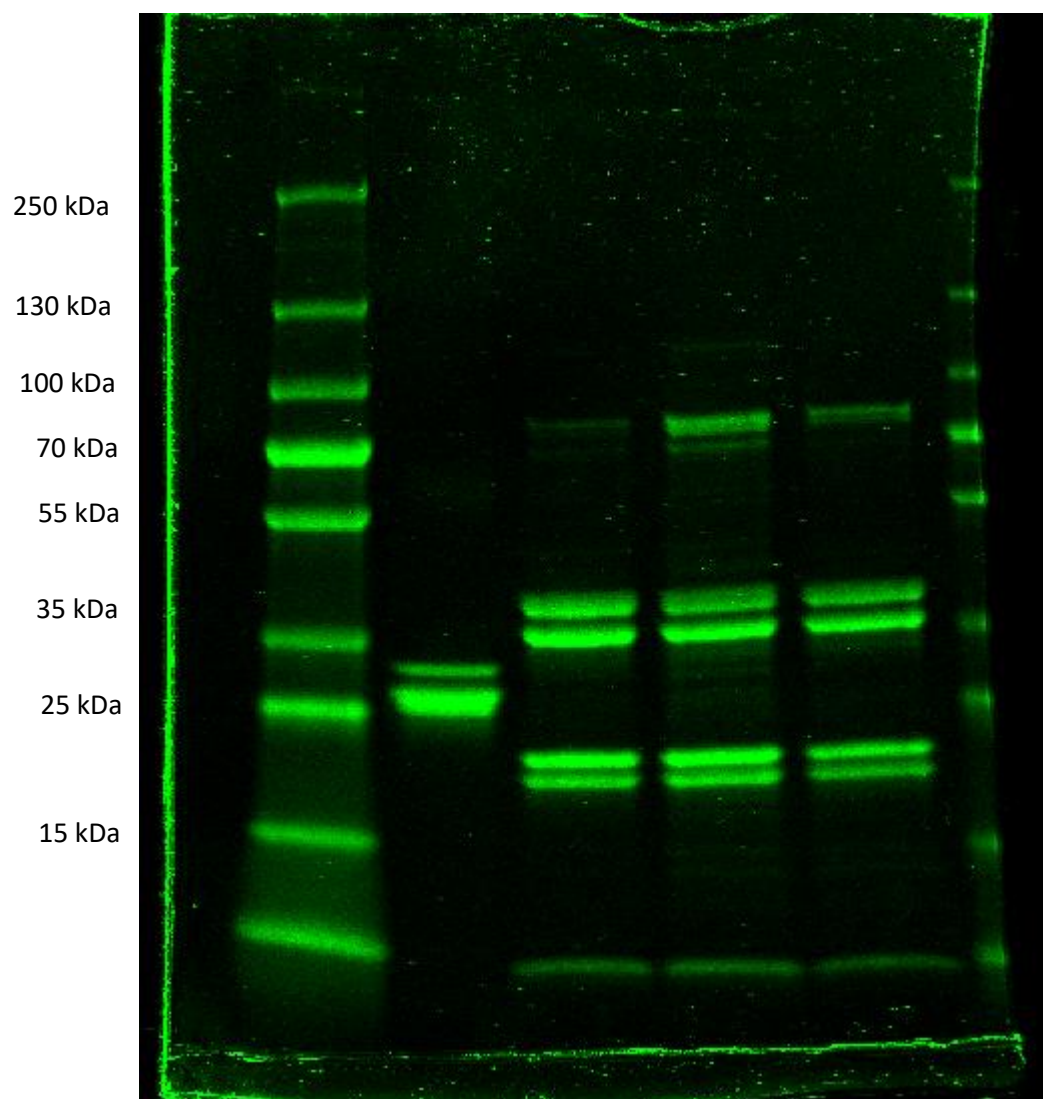

Supplement: Source Data Fig. 2 — Unprocessed western blots, gels and spot assays. [file 41594_2022_912_MOESM6_ESM.pdf]

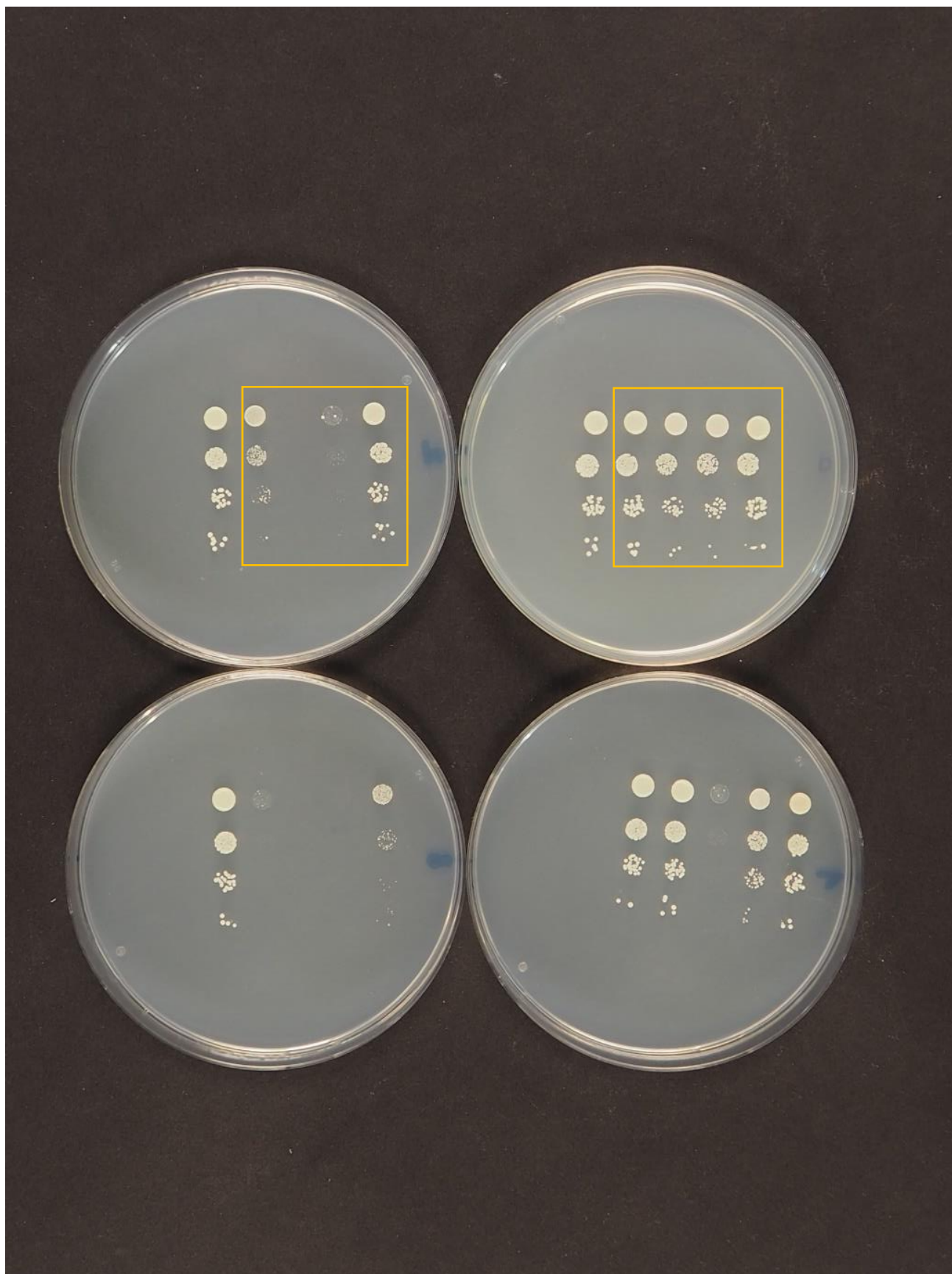

130 kDa

100 kDa

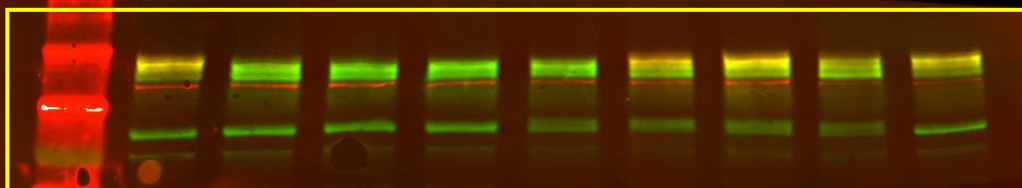

250 kDa

130 kDa

100 kDa

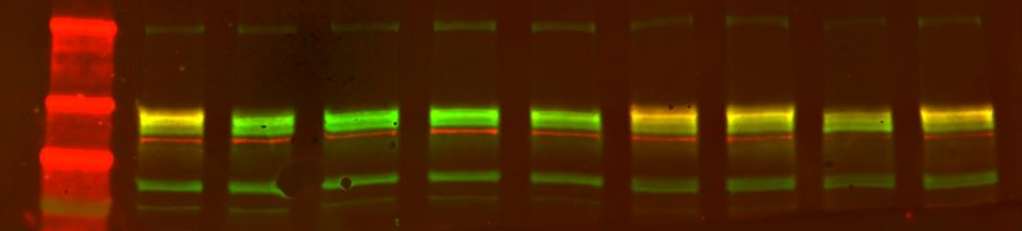

250 kDa

130 kDa

100 kDa

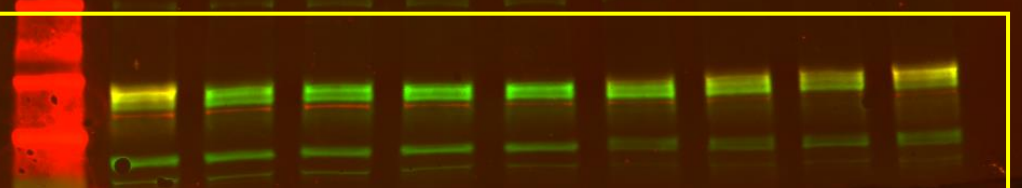

250 kDa

130 kDa

100 kDa

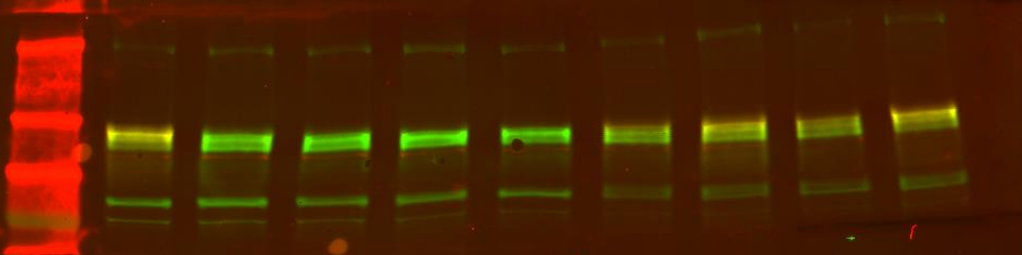

250 kDa

130 kDa

100 kDa

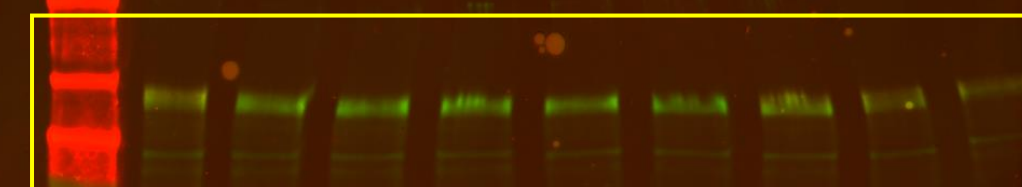

250 kDa

130 kDa

100 kDa

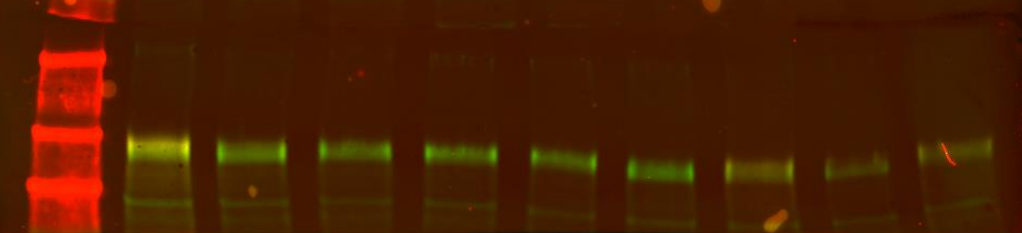

250 kDa

130 kDa

100 kDa

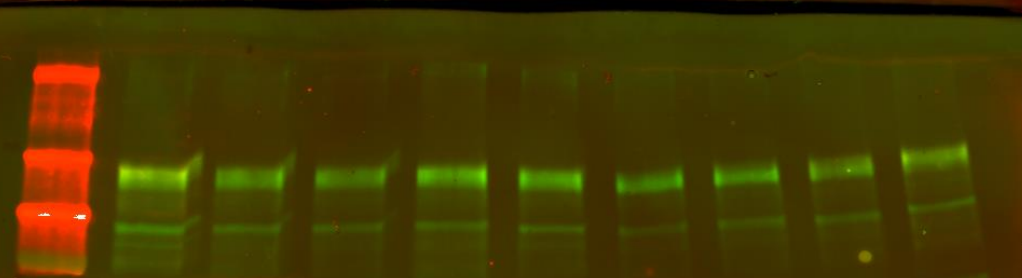

250 kDa

130 kDa

100 kDa

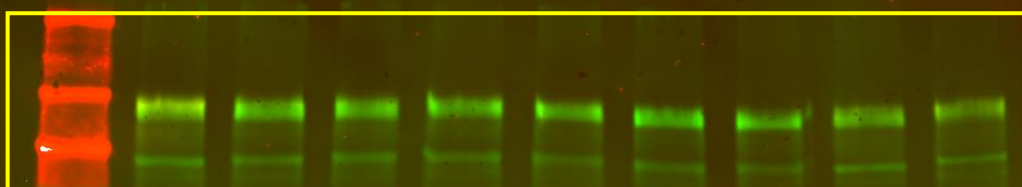

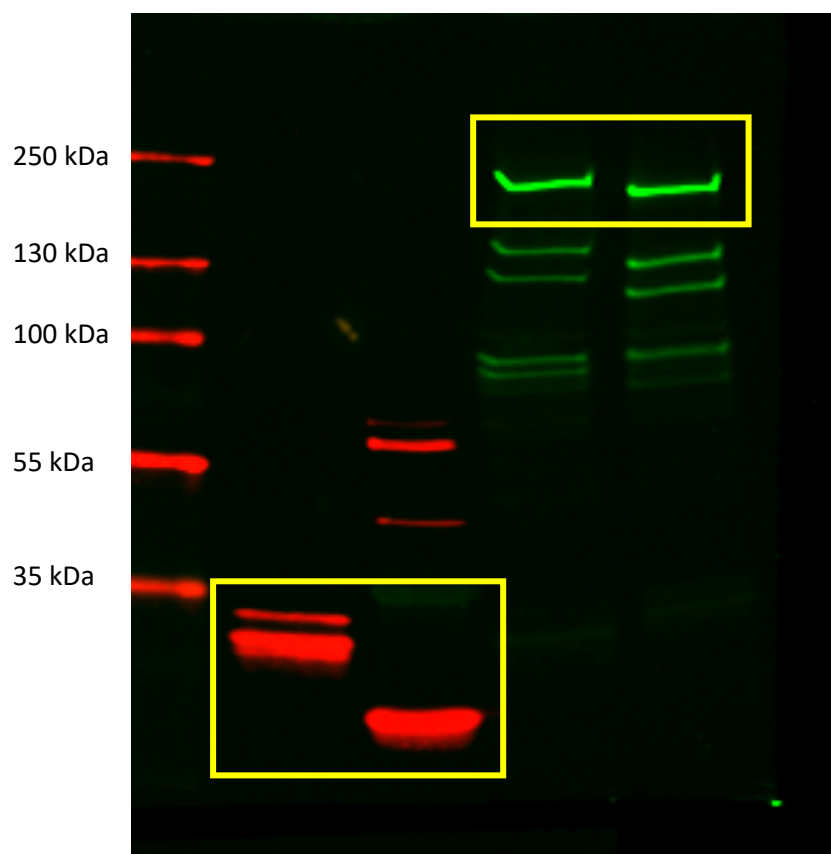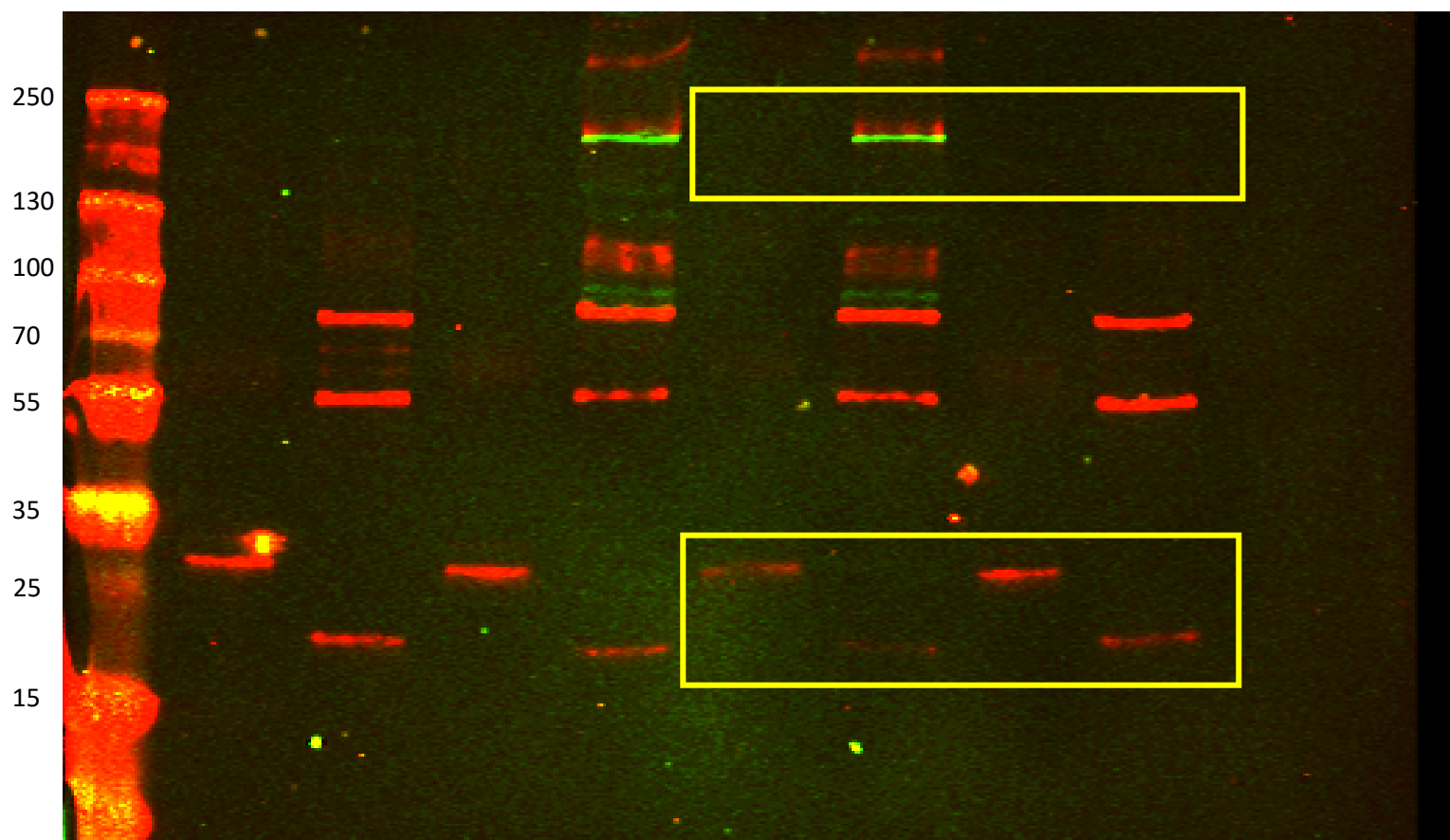

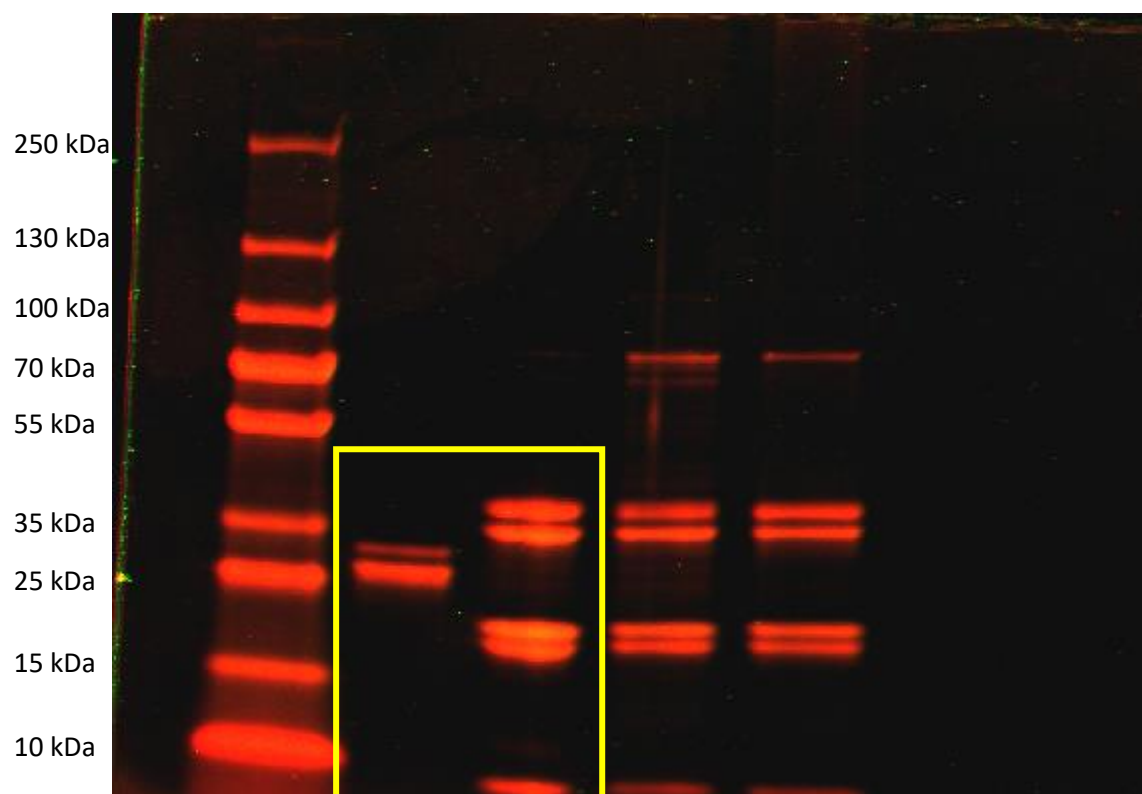

Supplement: Source Data Fig. 6 — Unprocessed western blot and spot assays. [file 41594_2022_912_MOESM9_ESM.pdf]

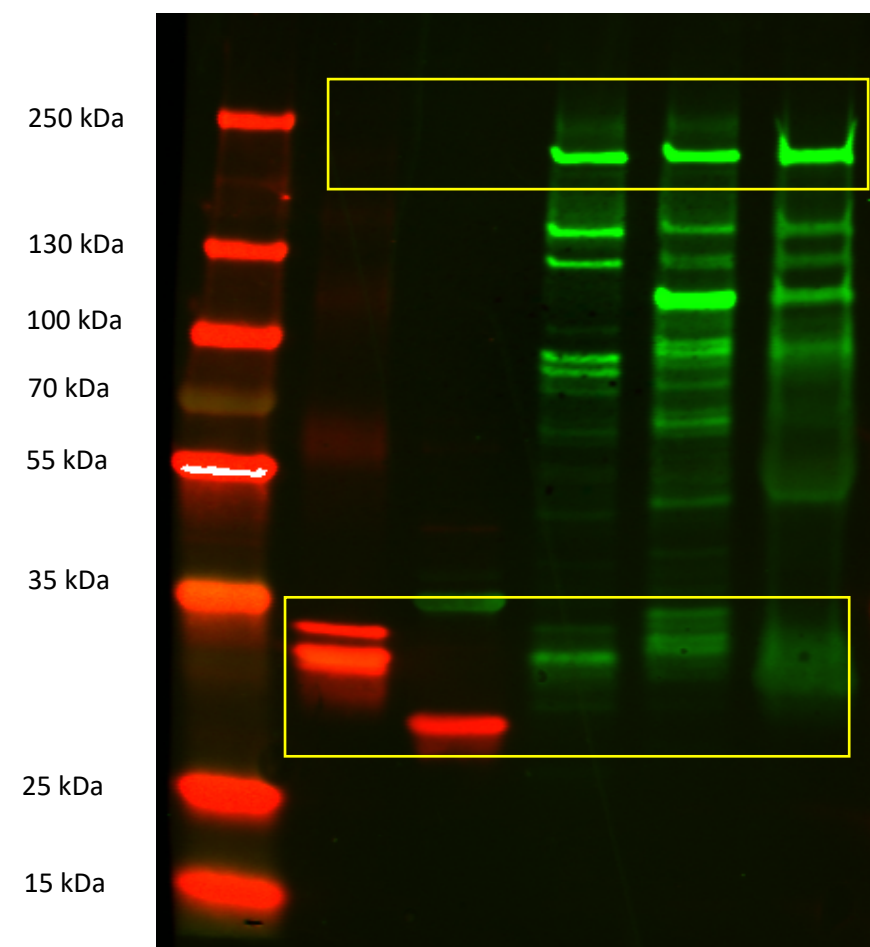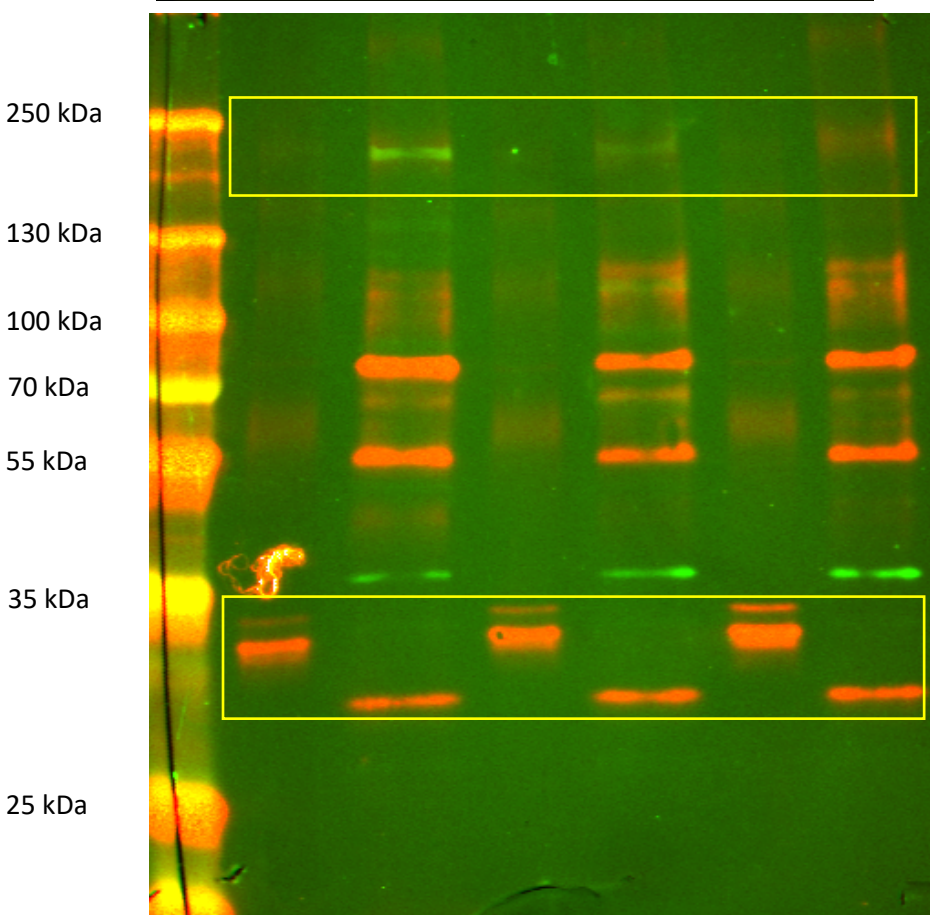

Supplement: Source Data Fig. 7 — Unprocessed western blot. [file 41594_2022_912_MOESM10_ESM.pdf]

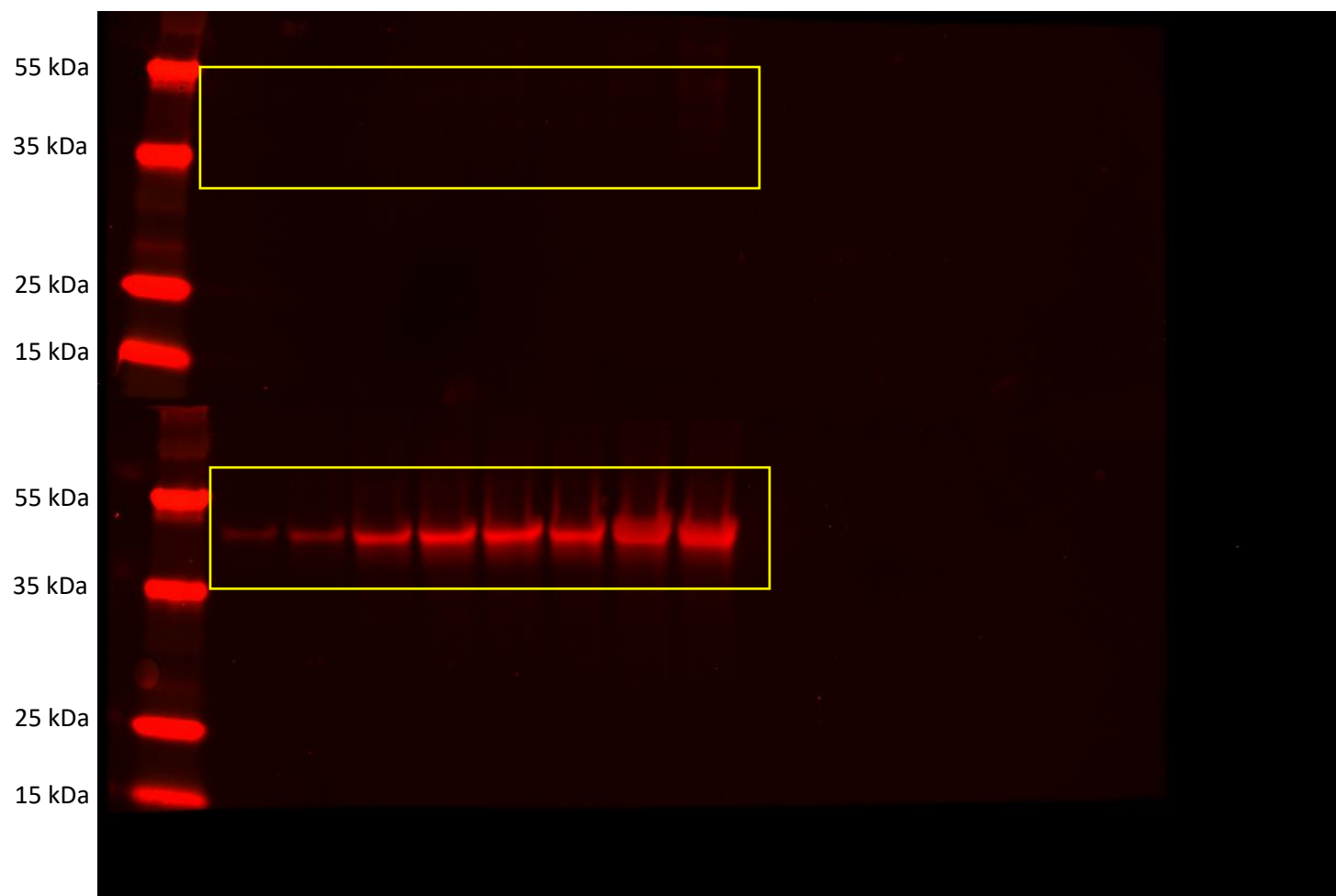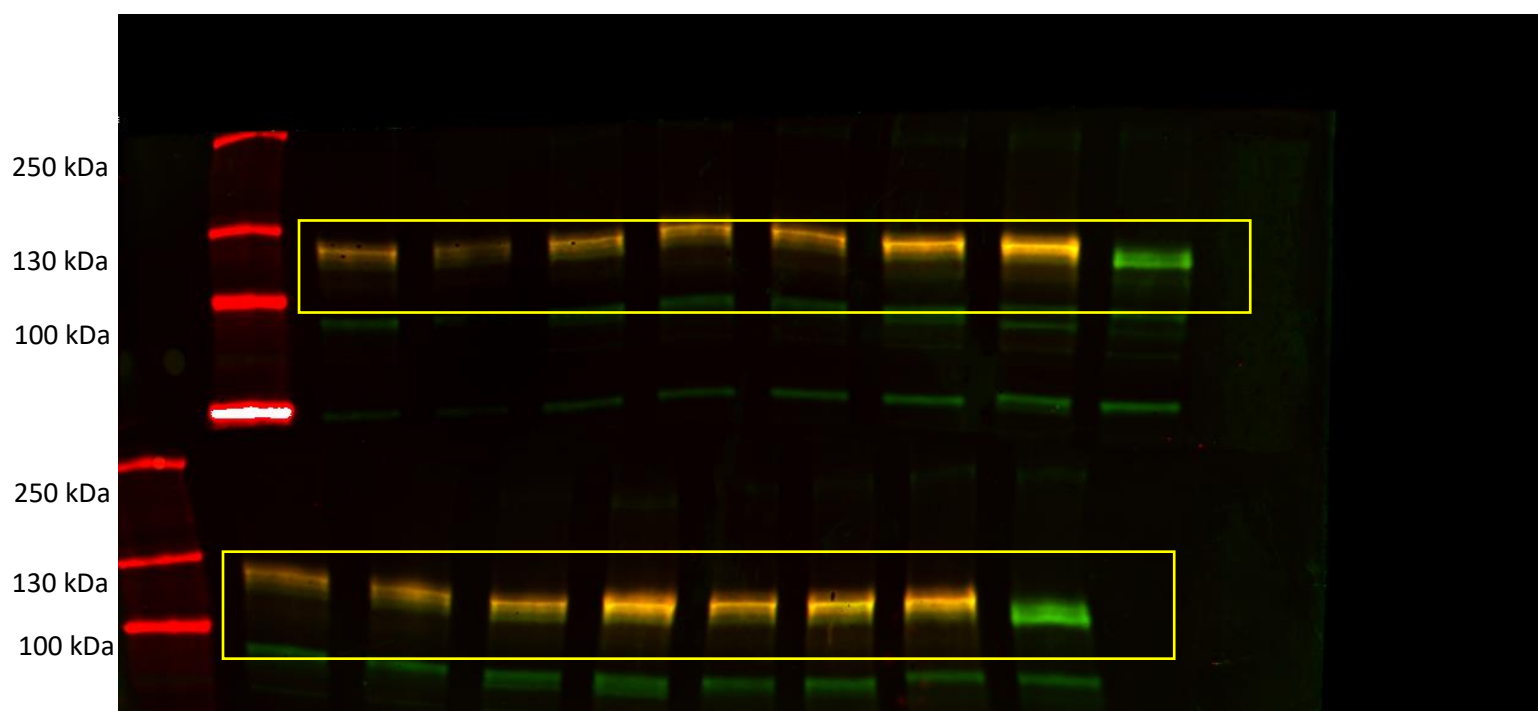

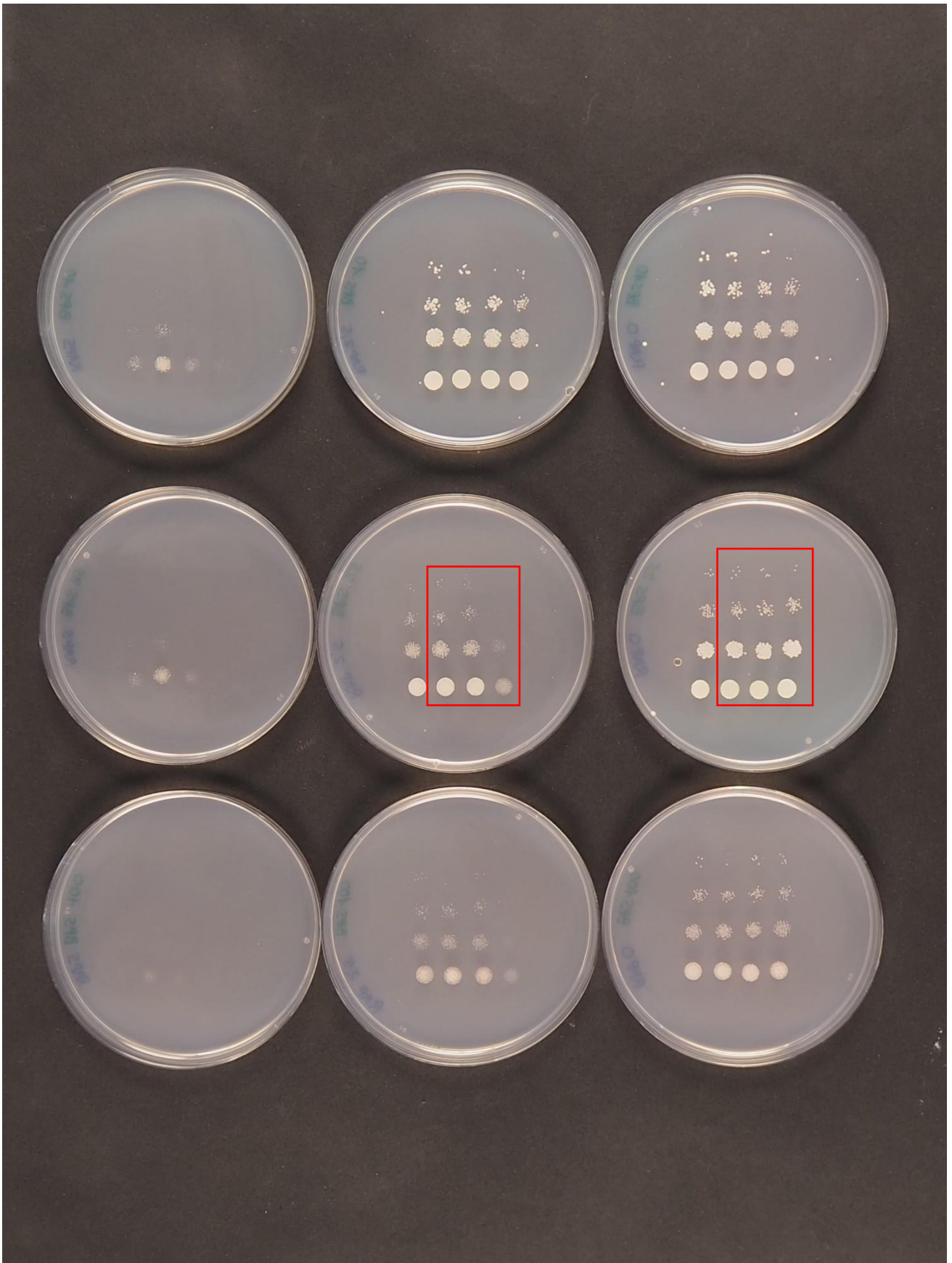

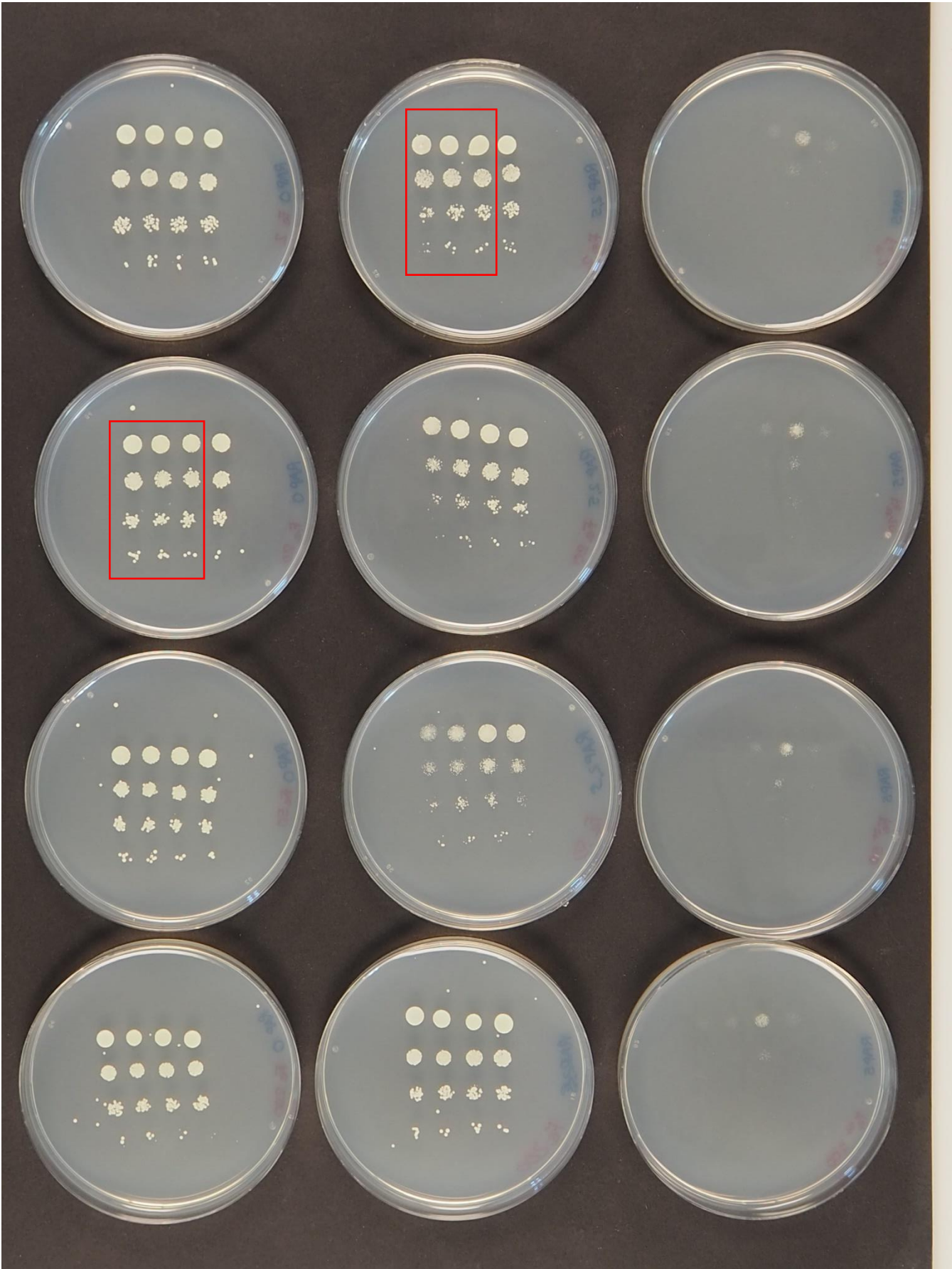

Supplement: Source Data Extended Data Fig. 1 — Unprocessed western blot and spot assays. [file 41594_2022_912_MOESM12_ESM.pdf]

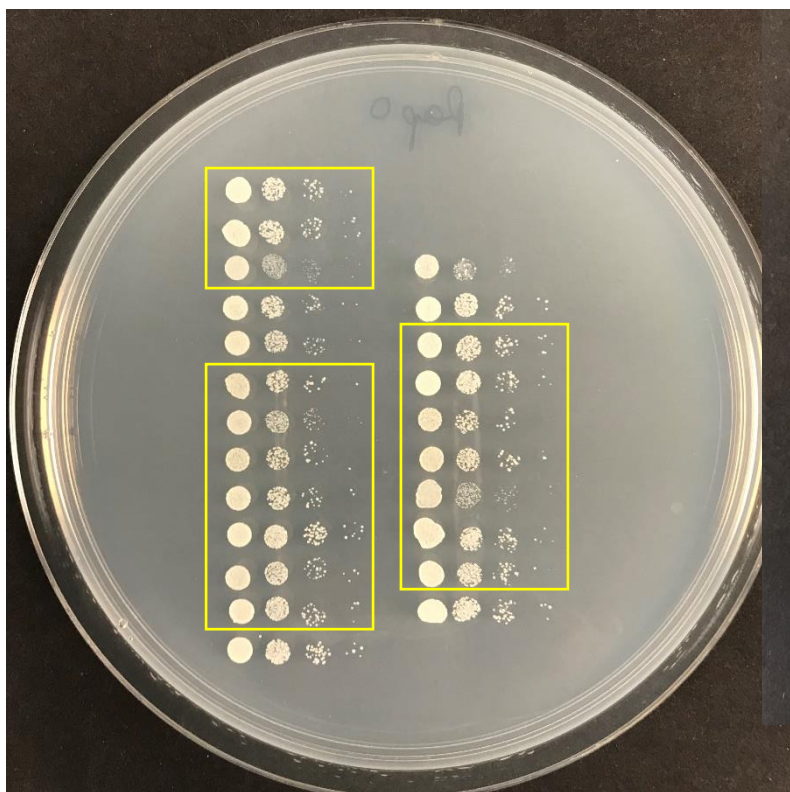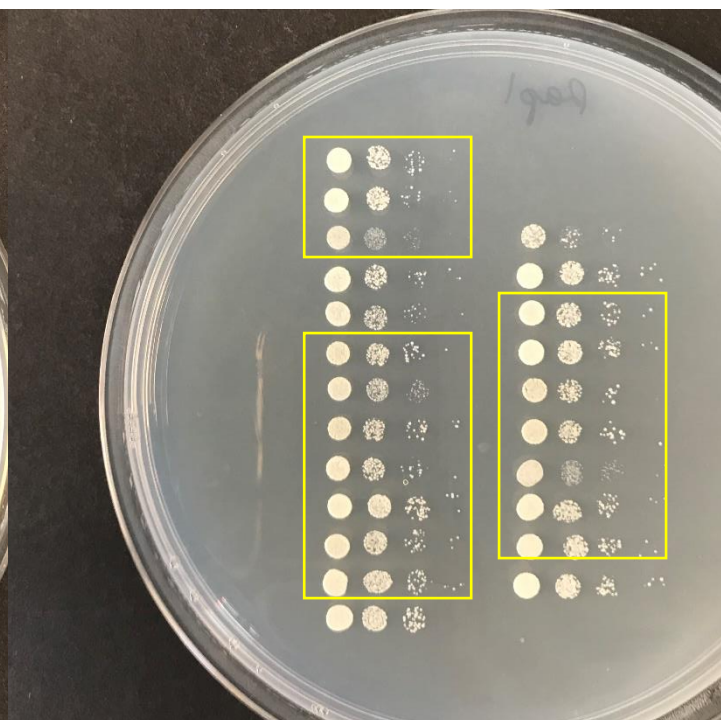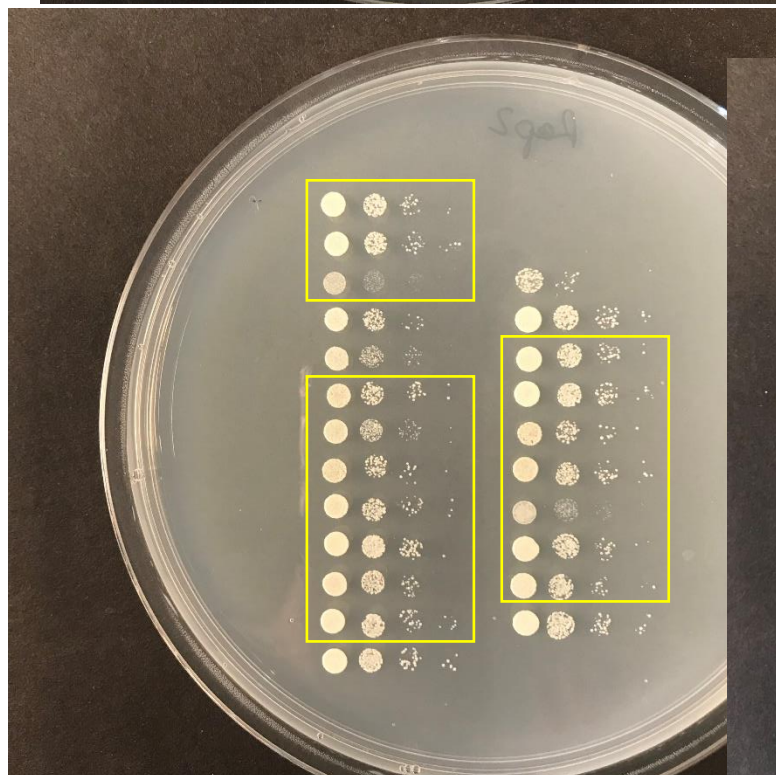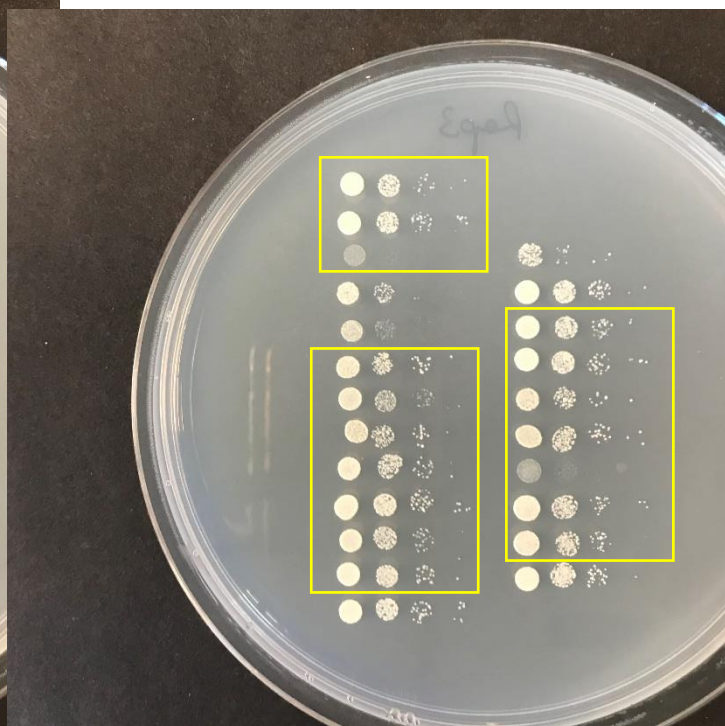

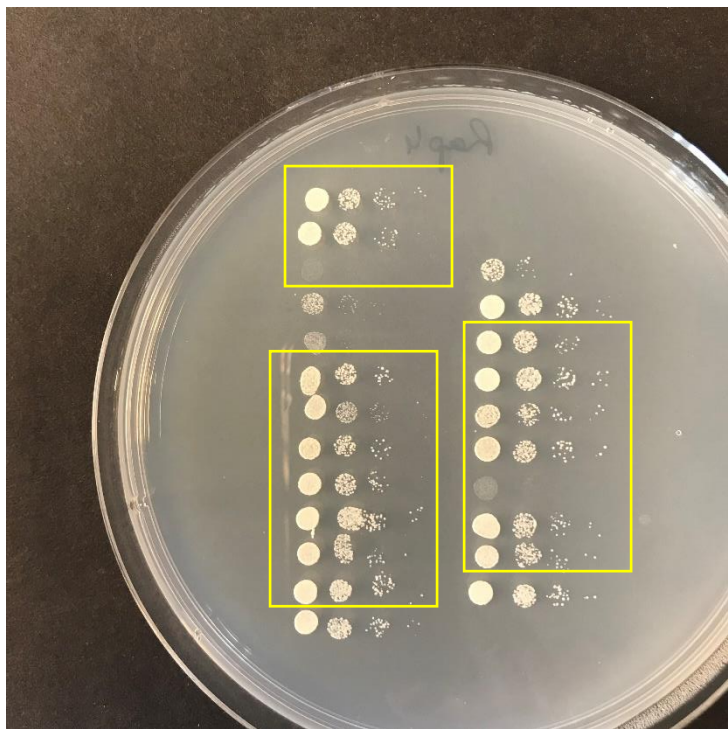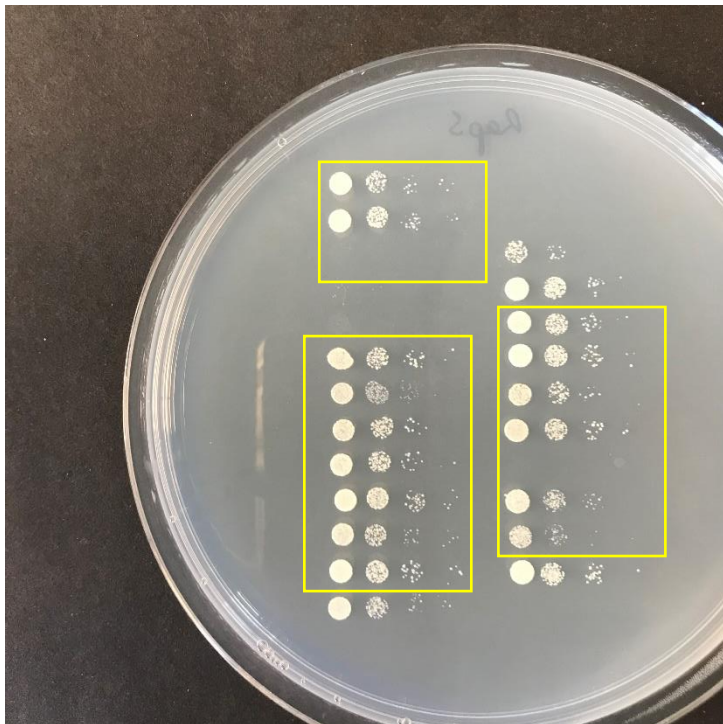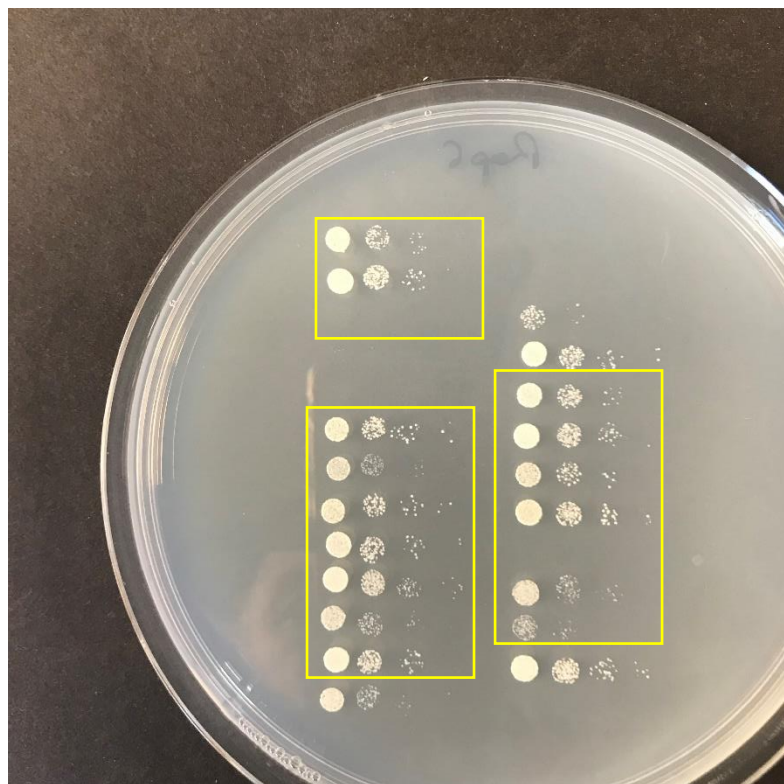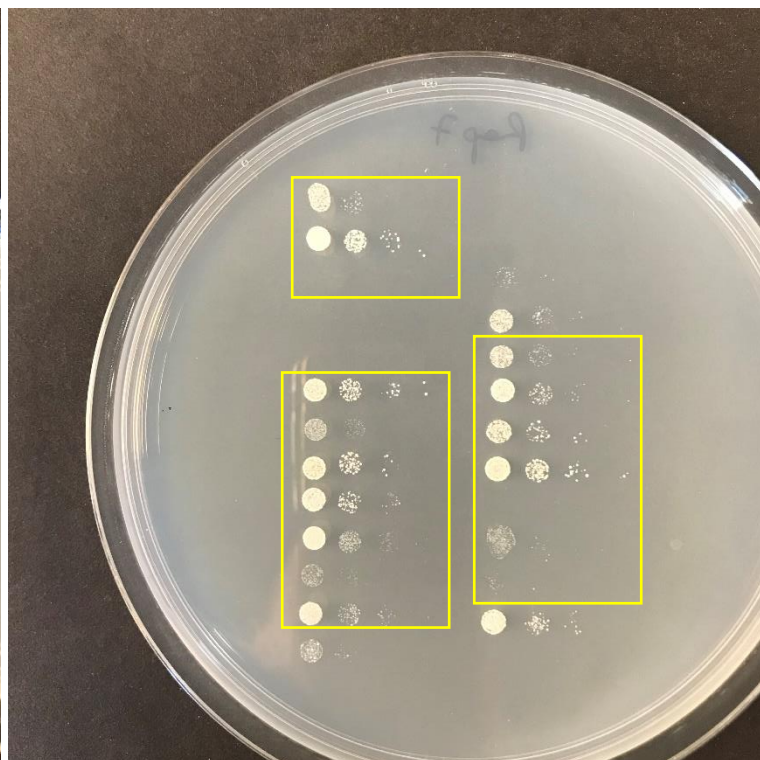

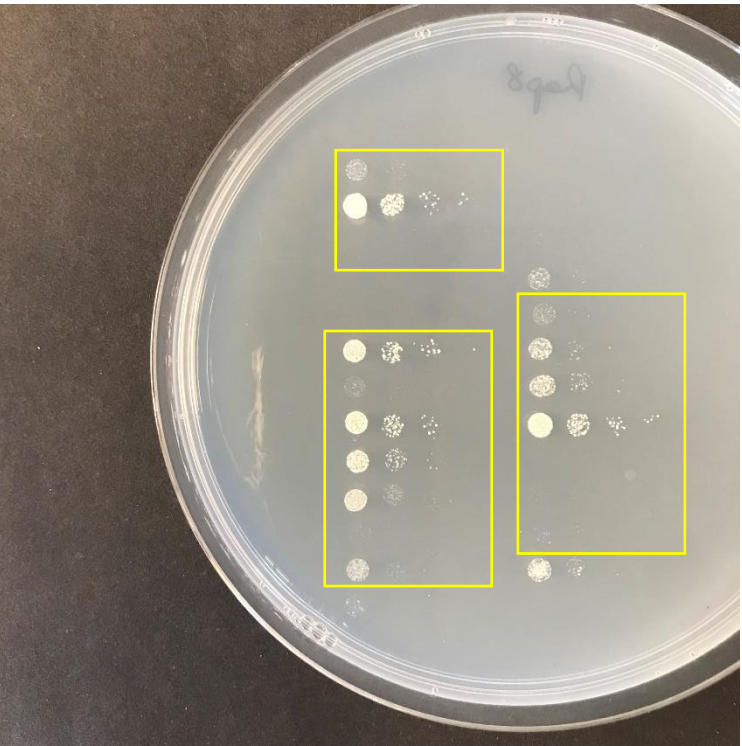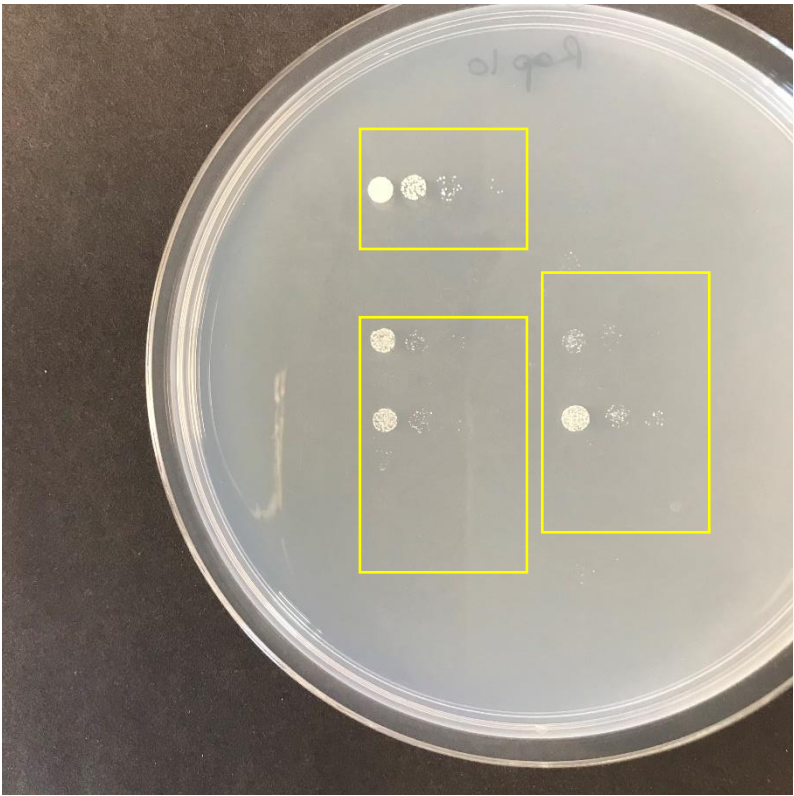

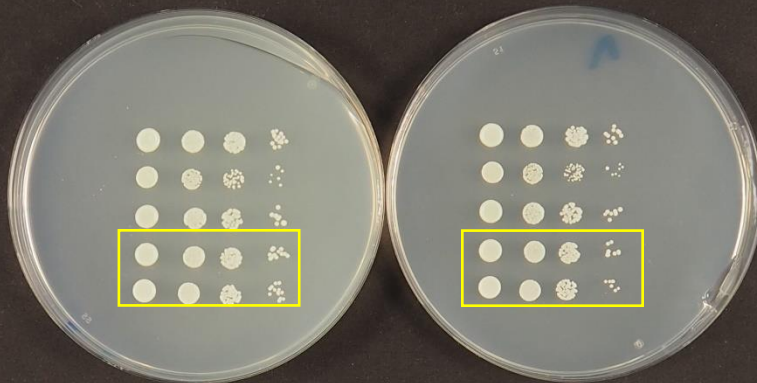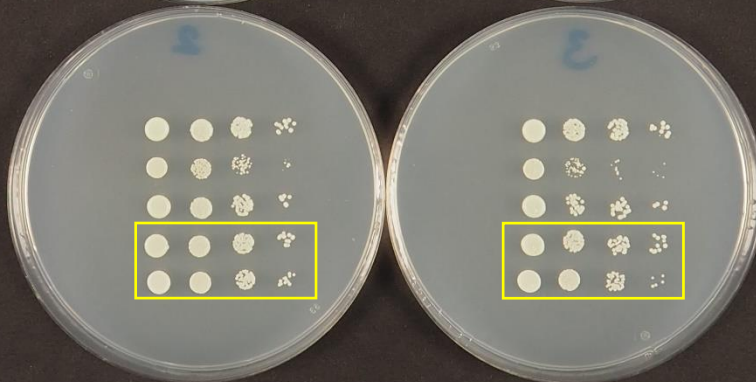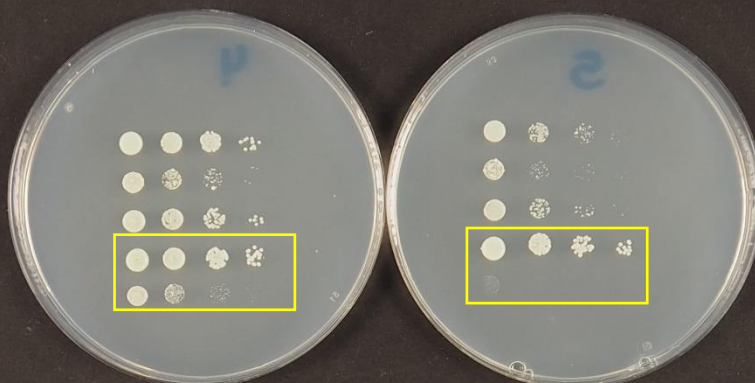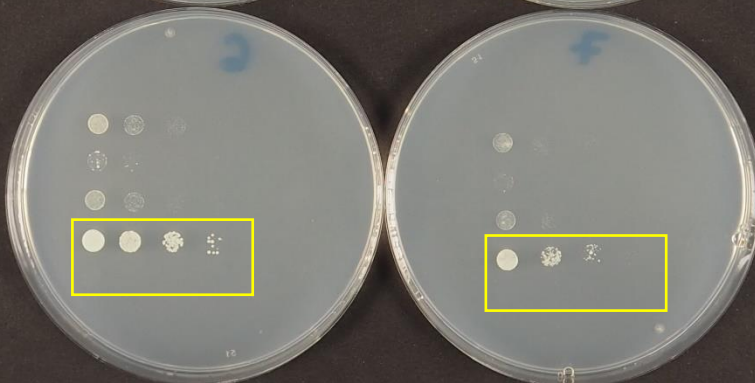

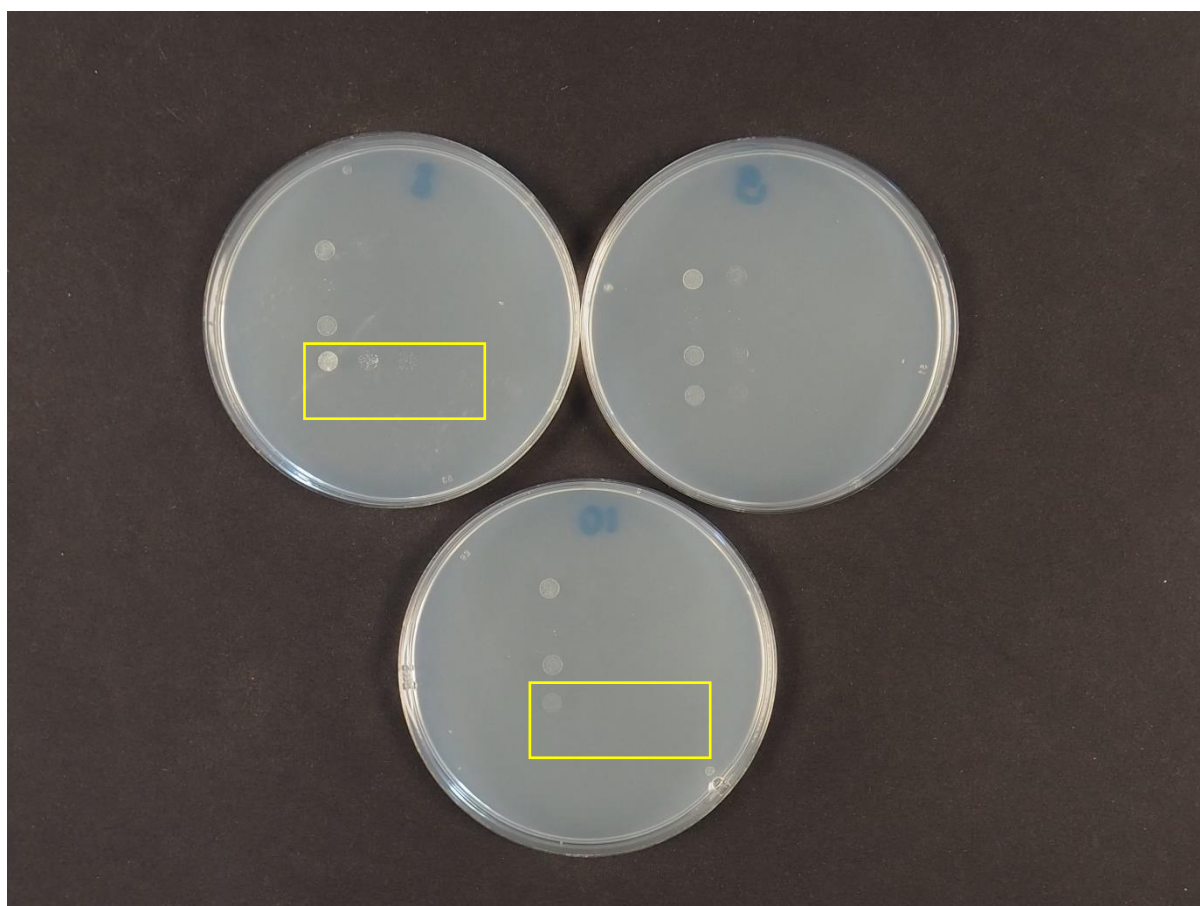

Supplement: Source Data Extended Data Fig. 6 — Unprocessed spot assays. [file 41594_2022_912_MOESM15_ESM.pdf]

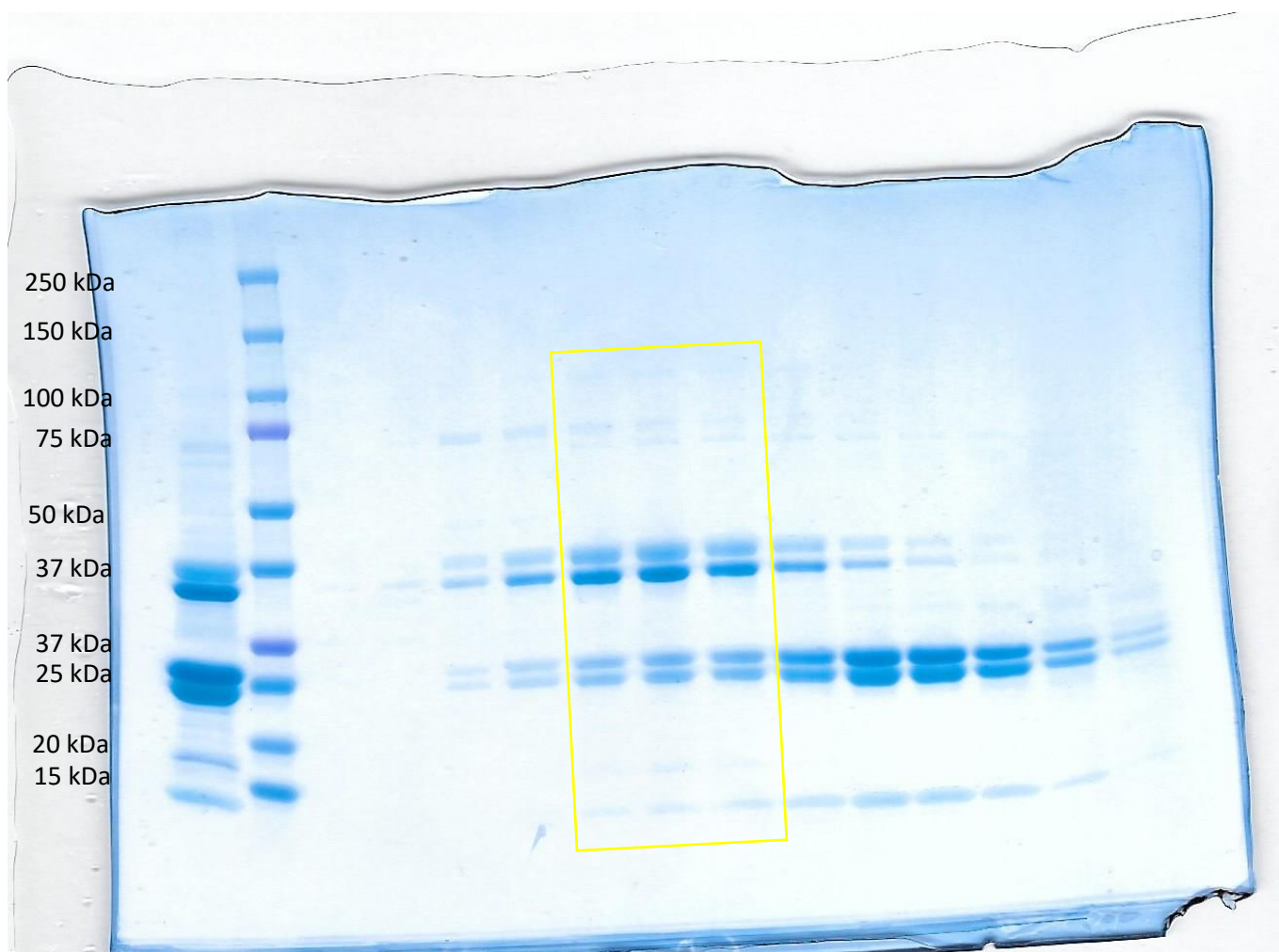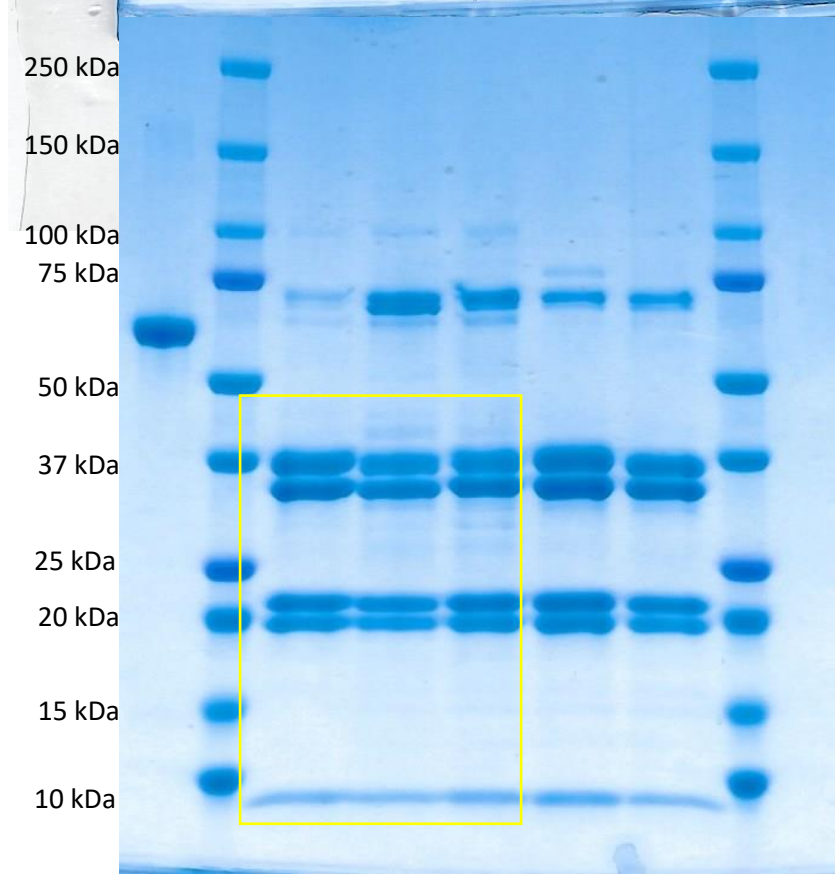

Supplement: Source Data Extended Data Fig. 7 — Unprocessed gel. [file 41594_2022_912_MOESM17_ESM.pdf]

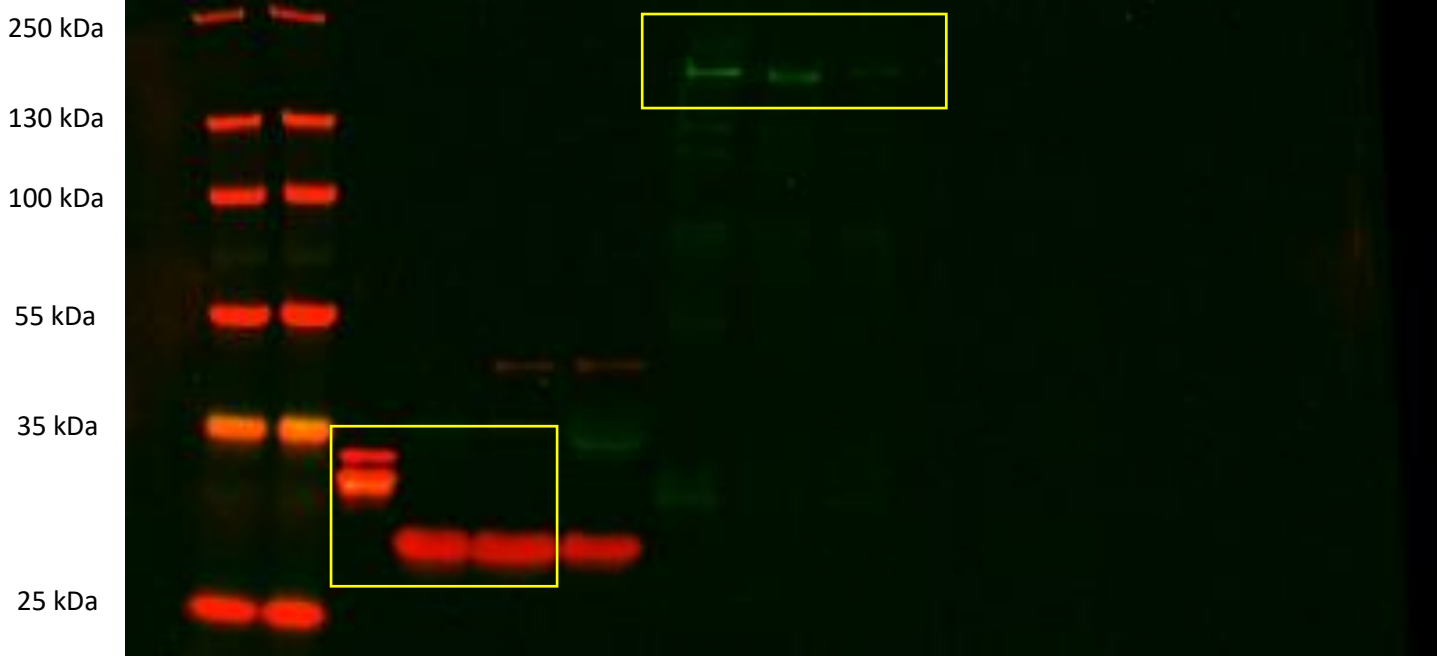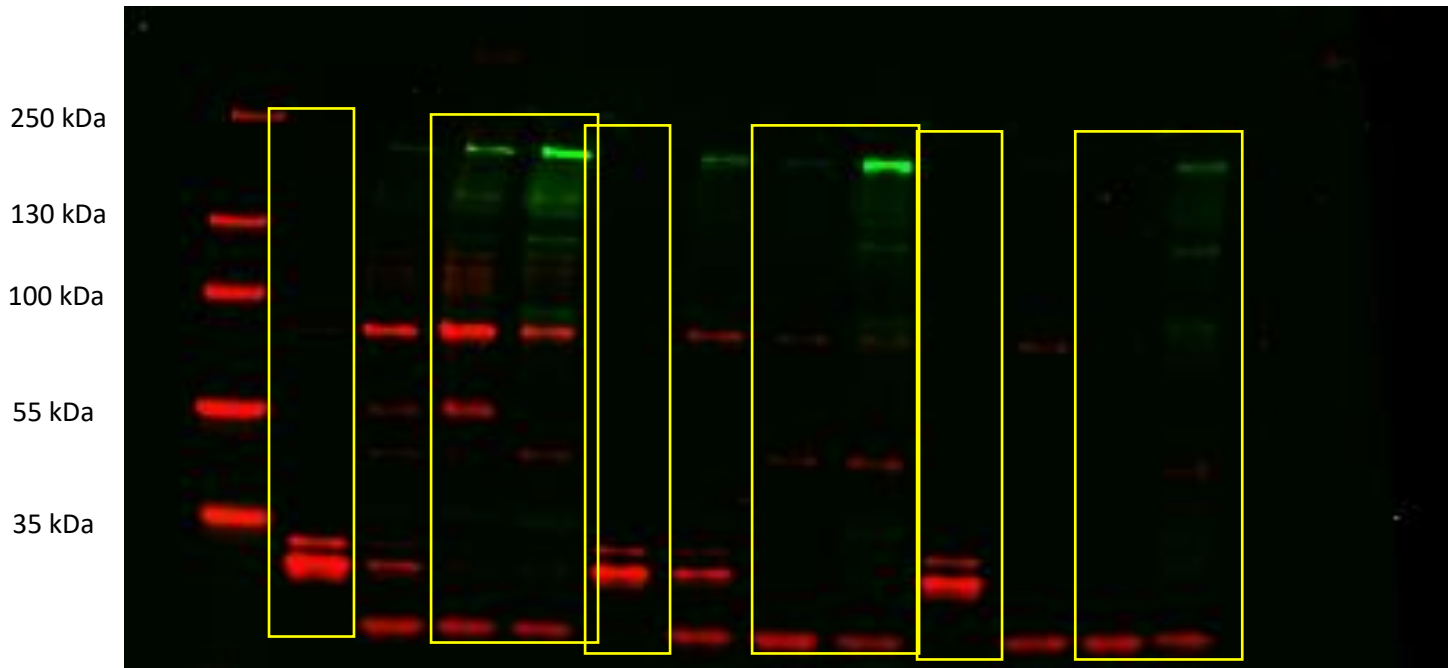

Supplement: Source Data Extended Data Fig. 8 — Unprocessed western blot. [file 41594_2022_912_MOESM19_ESM.pdf]
